# Repellent activity of essential oils to the Lone Star tick, *Amblyomma americanum*

**DOI:** 10.1186/s13071-024-06246-0

**Published:** 2024-05-06

**Authors:** Anais Le Mauff, Edmund J. Norris, Andrew Y. Li, Daniel R. Swale

**Affiliations:** 1 Emerging Pathogens Institute, Department of Entomology and Nematology, University of Florida, 2055 Mowry Road, PO Box 100009, Gainesville, FL 32610 USA; 2 Center for Medical, Agricultural, and Veterinary Entomology, United States Department of Agriculture, Agricultural Research Service, Gainesville, FL 32608 USA; 3 Invasive Insect Biocontrol & Behavior Laboratory, United States Department of Agriculture, Agricultural Research Service, Beltsville, MD 20705 USA

## Abstract

**Background:**

The Lone Star tick, *Amblyomma americanum* is important to human health because of a variety of pathogenic organisms transmitted to humans during feeding events, which underscores the need to identify novel approaches to prevent tick bites. Thus, the goal of this study was to test natural and synthetic molecules for repellent activity against ticks in spatial, contact and human fingertip bioassays.

**Methods:**

The efficacy of essential oils and naturally derived compounds as repellents to *Am. americanum* nymphs was compared in three different bioassays: contact, spatial and fingertip repellent bioassays.

**Results:**

Concentration response curves after contact exposure to 1R-trans-chrysanthemic acid (TCA) indicated a 5.6 μg/cm^2^ concentration required to repel 50% of ticks (RC_50_), which was five- and sevenfold more active than DEET and nootkatone, respectively. For contact repellency, the rank order of repellency at 50 μg/cm^2^ for natural oils was clove > geranium > oregano > cedarwood > thyme > amyris > patchouli > citronella > juniper berry > peppermint > cassia. For spatial bioassays, TCA was approximately twofold more active than DEET and nootkatone at 50 μg/cm^2^ but was not significantly different at 10 μg/cm^2^. In spatial assays, thyme and cassia were the most active compounds tested with 100% and 80% ticks repelled within 15 min of exposure respectively and was approximately twofold more effective than DEET at the same concentration. To translate these non-host assays to efficacy when used on the human host, we quantified repellency using a finger-climbing assay. TCA, nootkatone and DEET were equally effective in the fingertip assay, and patchouli oil was the only natural oil that significantly repelled ticks.

**Conclusions:**

The differences in repellent potency based on the assay type suggests that the ability to discover active tick repellents suitable for development may be more complicated than with other arthropod species; furthermore, the field delivery mechanism must be considered early in development to ensure translation to field efficacy. TCA, which is naturally derived, is a promising candidate for a tick repellent that has comparable repellency to commercialized tick repellents.

**Graphical Abstract:**

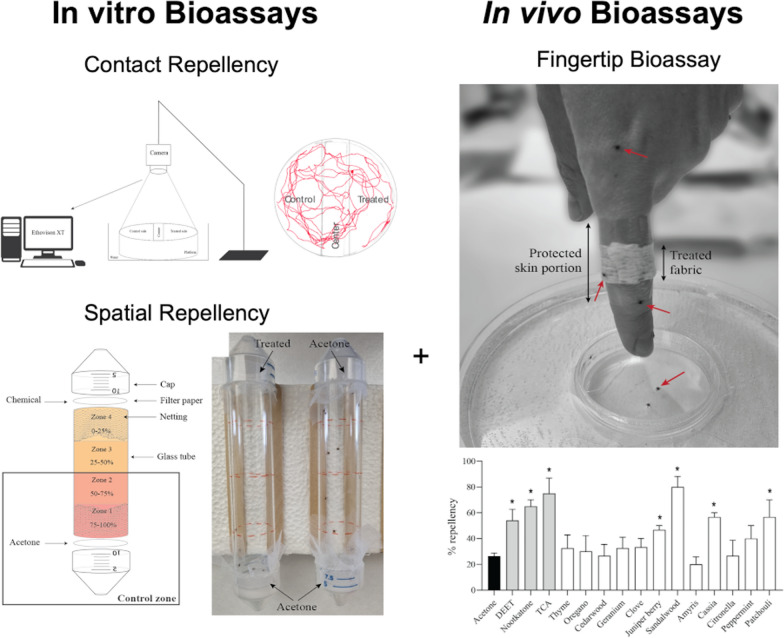

**Supplementary Information:**

The online version contains supplementary material available at 10.1186/s13071-024-06246-0.

## Background

Ticks are a significant threat to animal and human health because they vector a variety of pathogenic microorganisms that cause disease. The Lone Star tick (*Amblyomma americanum*) in the USA is a major concern as it is known to vector a variety of pathogens, is one of the most aggressive biting ticks for humans [1–3], and there is a growing body of work that suggests bites from *Am. americanum *give rise to alpha-gal syndrome or red meat allergy [4, 5].  Thus, there is an increased need for the development of mechanisms that can prevent tick bites to reduce transmission of tick vectored pathogens or allergies induced from tick bites. Appropriate use of repellents is recommended by the US Center for Disease Control (CDC) to reduce or prevent tick bites and horizontal transmission of tick-borne pathogens to humans, and the use of personal protectants to prevent tick bites is moderately accepted across the general population [6–8]. However, the chemical diversity of tick repellents available to the consumer remains low.

N,N-diethyl-*m*-toluamide, known as DEET, is considered the ‘gold standard’ for mosquito repellents due to its high repellency across different repellent assays and positive selectivity profile [9, 10]. Interestingly, tick repellency to DEET is variable as some studies report high efficacy at 10% [11] while other studies report 10% DEET is not highly repellent to *Ixodes* or *Amblyomma *[12, 13]. Other natural or synthetic repellents, such as picaridin, IR3535, lemon eucalyptus oil, *p*-menthane-3,8-diol (PMD) and 2-undecanone, have been shown to have repellent properties to ticks, but their effectiveness and/or duration of effectiveness is less than optimal [14, 15]. A review of the literature shows differences in efficacy of tick repellents between studies that is often attributed to differences in tick species. For instance, 20% DEET repels nearly 100% of *Am. americanum* for a prolonged period with an approximate concentration to repel 50% of ticks (RC_50_) of 10% [16, 17] whereas Semmler [13] reported high concentrations of DEET are needed to repel *Ixodes ricinus* and *Dermacentor reticulatus* [13]. While it is likely the different efficacies of repellents are partly due to species differences, the assays used are highly variable between studies and may also contribute to differences in repellency. A variety of repellent bioassays have been designed to test the efficacy of novel tick repellents that range from in vitro bioassays, such as the petri dish assay [18–21], vertical filter paper bioassay [21–27] and carousel assay [28], to in vivo assays that measure tick engagements or climbing, such as the fingertip or forearm bioassay [26, 29–31]. The experimental design is particularly relevant for ticks as the host-seeking, or questing, behavior of ixodid ticks is species and life stage specific [32]. Furthermore, the experimental design of in vitro assays is unlikely to account for external stimuli that are relevant to questing behavior [33] and raises the question of how well in vitro assays correlate to repellent efficacy to questing ticks in the field. Although there have been some efforts to compare repellency bioassay methods for ticks [30, 31], there is still no consensus for the most appropriate bioassay to test for tick repellency.

Considering (1) the lack of consensus for the most appropriate bioassay to test for tick repellency, (2) lack of highly effective tick repellents and (3) shortened pipeline for commercialization for natural products [34–36], the goal of this study was to compare the efficacy of essential oils and naturally derived compounds as repellents to *Am. americanum* nymphs in three different bioassays: contact, spatial and fingertip repellent bioassays. We compared the tick repellency of 16 plant-extracted oils and one recently identified naturally derived mosquito repellent [37, 38], 1R-trans-chrysanthemic acid (TCA), to EPA registered tick repellents, DEET and nootkatone.

## Methods

### Chemicals

Acetone was used as a solvent for all assays and was purchased from Thermo Fisher Scientific (Pittsburgh, PA, USA). DEET (97%) and ( +)-nootkatone (≥ 99.0%, GC) were purchased from Sigma-Aldrich Chemical Co. (St Louis, MO, USA). Thyme (from *Thymus zygis*) oil, fennel (from *Foeniculum vulgare*) oil, lemon (from *Citrus limonum*) oil, black pepper (from *Piper nigrum*) oil and cedarwood (from *Cedarus deodora*) oil were obtained from Edens Garden (San Clemente, CA, USA). Lavender (from *Lavandula angustifolia*) oil and dill (from *Anethum graveolens*) oil were both obtained from Plant Therapy (Twin Falls, ID, USA). Other essentials oils tested, such as oregano, geranium, clove, amyris, patchouli, peppermint, citronella and juniper berry, were obtained from Berje, Inc. (Carteret, NJ, USA). We recognize oils can vary in purity and percent components; thus, we performed gas chromatography/mass spectrometry (GC/MS) to verify components of each batch of oil used to ensure purity and components of the oils were standard across all treatments.

### Gas Chromatography/Mass Spectrometry

Gas chromatography-mass spectrometry (GC/MS) analyses were performed on a Thermo Scientific (Waltham, MA, USA) Trace 1310 GC coupled with a Thermo Scientific ISQ7000 mass detector and equipped with a Thermo Scientific Trace Gold TG-5SILMS capillary column (30 mm, 0.25 mm inner diameter, 0.25 µm film thickness). The oven temperature program was initiated at 50 °C and held for 1 min before raising the temperature 3 °C/min to 300 °C, then holding for 10 min. He (99.9999%) was used as the carrier gas with a flow rate of 2.2 ml/min. The injector temperature was 250 °C with a split ratio of 1/50. Mass spectra were recorded at 70 eV with a mass range from *m/z* 33 to 550. Constituents were identified and declared if they represented at least 0.1% of the total volume of the plant oil. Constituents of each plant oil are featured in Additional file 1: Table S1.

### Ticks

*Amblyomma americanum* nymphs were purchased from the Oklahoma State University Tick Rearing Facility (Department of Entomology; Stillwater, OK, USA). Ticks were used in repellency bioassays 2–3 weeks after molting and were maintained in an incubator at 28˚C and 60% RH with 12:12 light:dark cycle in the Swale Laboratory (Emerging Pathogens Institute, University of FL) before being used in experimental assays. The supplier declared that all ticks used were free of all known pathogens.

### Repellency bioassays

#### Contact repellent bioassay

**Fig. 1:**
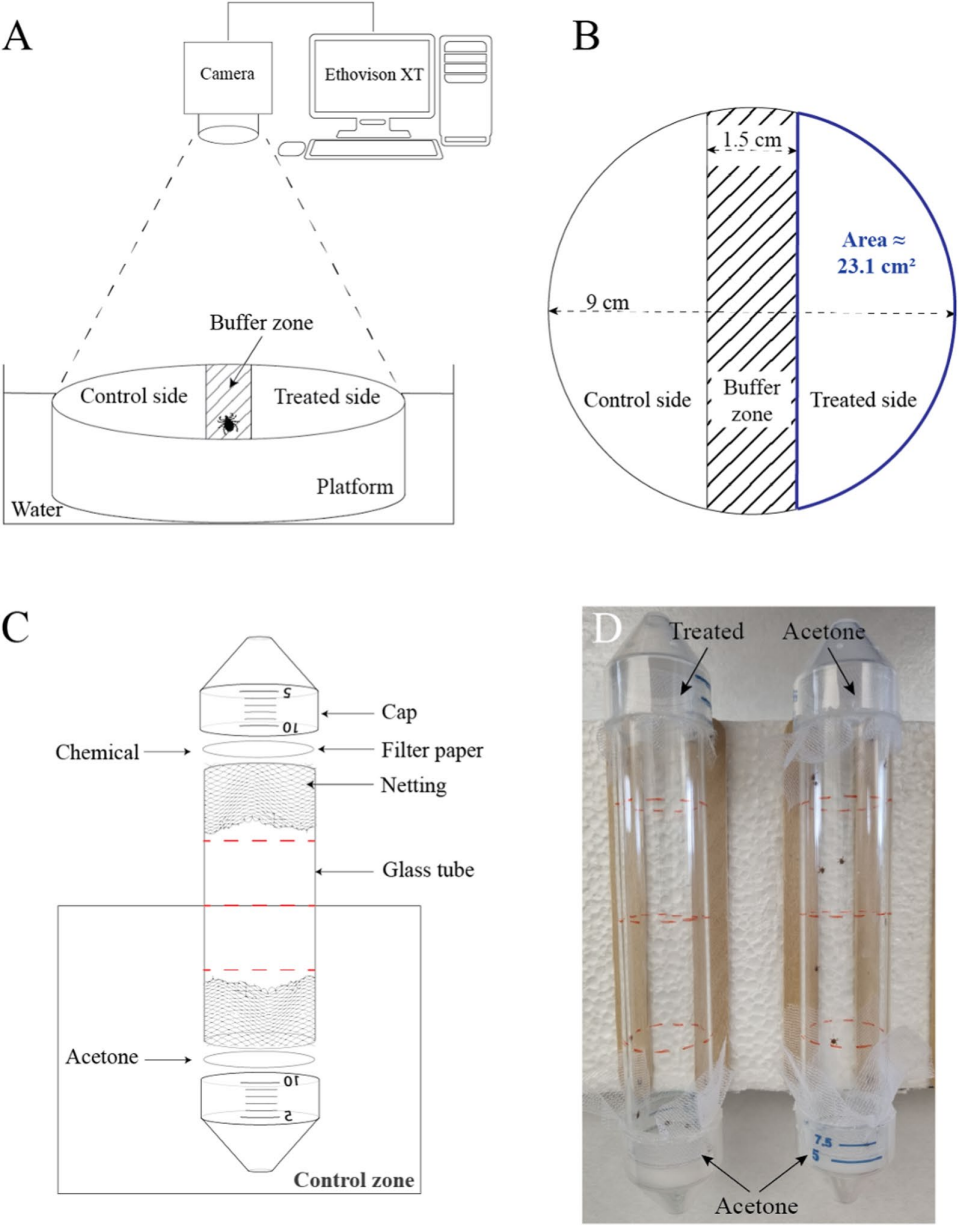
Design of in vitro repellent bioassays to determine potency of repellents to *Amblyomma americanum* nymphs. **A**–**B** Schematic overview (**A**) and design (**B**) used for the contact repellent assay. A polystyrene platform, on which the tick movements were recorded, is designed as shown in the picture with three distinct areas: the control side (treated with acetone), center (where ticks were placed at the start of the experiment) and treated zone (treated with chemical or with acetone for control trial). The platform is fixed in a container filled and surrounded by water to avoid tick escape. The camera is positioned at the top of the platform and linked to the Ethovision software, which allows analyzing the video obtained.** C** Drawing showing the design used for the spatial repellency of nymphs. The control zone (between the middle of the tube and control side) and different zones noted on the tube by red hash marks and the approximative percentage of repellency in each zone regarding the treated side (top of the tube). **D** Picture of tubes containing 10 nymphs that were positioned horizontally. The left tube is treated with TCA where ticks are located toward the acetone side of the tube, and the tube on the right is solvent control where ticks are evenly distributed throughout the tube

We assessed contact repellency or attractancy of compounds to *Am. americanum* nymphs in the afternoon hours (12 p.m. to 5 p.m.) with approximate temperature and relative humidity of 26 °C and 70% RH. We ranked compound potency by quantifying the movement of one tick placed on the buffer zone of a round polystyrene platform surrounded by water in which an untreated, treated and buffer zone were defined (Fig. 1A, B). Tick movement was tracked using the Ethovision XT video recording software and a Basler acA-1300-60gm camera (Noldus Information Technology Inc., Leesburg, VA, USA) mounted within an enclosed arena that blocked all external visual stimuli. Importantly, the equipment was mounted on rubber for vibration isolation to further reduce interference of tick movements from external stimuli. The surface of the polystyrene platform was protected using a bench protector layer (Thermo Fisher Scientific) on which the treated and control filter papers were placed before the experiment. The substrate was cut from 9-cm-diameter round filter papers (Themo Fisher Scientific) following the dimensions shown in Fig. 1A and B. Filter papers were treated with 200 μl acetone for the control side and 200 μl treatment solution for the treated side. After a drying time of 10 min on the bench, the two filter papers were attached to the platform, and the tick was placed on the buffer zone (1.5 cm width) of the platform. Movements were recorded over a 10-min period, and ticks were not reused for any other repellency experiment described in this study.

Bioassays with different repellents were conducted in a randomized order, and a solvent control group was utilized on each day and for each different repellent. For each compound, several concentrations were used to compare their repellency efficacy. DEET and nootkatone were tested at 1, 10, 50 and 100 μg/cm^2^, whereas TCA was tested at 0.25, 1, 5, 10, 25 and 50 μg/cm^2^, which enabled the generation of a concentration-response curve and the determination of the concentration to repel 50% of ticks (RC_50_). Essential oils (i.e. geranium, oregano, cedarwood, thyme, clove, juniper berry, amyris, cassia, citronella, peppermint, patchouli, fennel, lemon, dill, black pepper and lavender) were tested at 10 and 50 μg/cm^2^.

The percentage of time spent in each zone on the platform (i.e. control and treated zones) was obtained by dividing the time spent in each zone by the total time of the experiment and multiplied by 100 to obtain a percentage. The percentage of time spent in the buffer zone, used to place the ticks at the start of the experiment, was not included in the calculation of the repellent effect of treatment tested. Thus, the percentage of presence in the control zone (or % in control zone) was determined with the following formula: 100 × (total time in control zone/(total time in treated zone + total time in control zone)). Importantly, all ticks moved from the buffer zone during the recording.

#### Spatial repellent bioassay

The spatial repellent assay was based on modifications of the mosquito repellent assay described by Jiang and colleagues (2019) [39]. The behavior of *Am. americanum* nymphs was observed using a horizontal device that allowed ticks to freely move inside a glass tube during a defined period of time. Double mesh netting was placed at each end of the glass tube (length: 12.5 cm, outer diameter: 2.5 cm, TriKinetics, Waltham, MA, USA) and was held in place with the bottom end of a 50-ml centrifuge tube (Falcon™, Corning Inc., Corning, NY, USA) as shown in Fig. 1C and 1D. Six holes were drilled in the conical caps to avoid air saturation inside glass tubes. All tubes were placed on a polystyrene platform (with wooden sticks glued to the platform to keep the tubes from rolling) and inside a plastic container where the temperature and humidity were maintained at 26 ± 1 °C and 75 ± 5%, respectively. Glass tubes were discarded after termination of the experiment and were not reused.

Ten *Am. americanum* nymphs were introduced inside each glass tube and were allowed to equilibrate to the new environment for 20 min prior to initiating the experiment. During this equilibration period, round filter papers (diameter 2.5 cm, grade 1 Whatman, Sigma Aldrich Chemical Co.) were treated with 50 μl of either solvent (acetone) control or treatment solutions. The treated filter papers were dried for 10 min prior to being placed inside the conical cap. Treated papers were approximately 50 mm away from the mesh netting to prevent tick contact with chemicals. The distance between the control side netting and treated side netting was the length of the glass tube (12.5 cm). From this, we defined that the ticks were repelled if they were located within the control half of the chamber, which was within 6.25 cm from the acetone-treated filter paper to the midline of the glass chamber. The location of each tube on the polystyrene platform and the control or treatment ends were randomly selected for each replication. Control treatments consisted of filter papers treated with acetone only on both ends of the tube (Fig. 1D). For chemical treatments, one side of the tube was treated with acetone and the other with the putative repellent dissolved in acetone (Fig. 1D). For DEET and nootkatone, we tested 1, 10, 50 and 100 μg/cm^2^. Concentrations tested for geranium, oregano, cedarwood, thyme, clove, juniper berry, amyris, cassia, citronella, peppermint, patchouli, fennel, lemon, dill, black pepper and lavender oils were 1 and 10 μg/cm^2^. Concentrations tested for TCA were 1, 10 and 50 μg/cm^2^. The percentage of tick presence in the control zone was calculated with the following formula: 100 × (number of ticks in the control area)/(total number of ticks), and tick positions were recorded at 15 min, 30 min, 1 h and 2 h. Six replicates were performed for each treatment and each concentration where each replicate contained the repellency from ten individuals. The number of tick control groups was higher than that of ticks tested with chemical because one control group was done for every replicate to enabled paired statistical analysis (*n* = 230 control nymphs).

#### Fingertip bioassay

Human fingertips were used to test tick repellency under the University of Florida Institutional Review Board (IRB) approved protocol (IRB202301534) and was modified from the work of Carroll et al. [29]. A portion of skin was first protected by a clear adhesive bandage which covered the joint between the second and the third phalanx up to the middle of the third phalanx of the left forefinger. A 100-μl volume of solvent (acetone) or solution was applied on a piece of gauze fabric (1.5 × 6.5 cm cut from non-woven wound pad, General Medi®) and left to dry at RT for 10 min. The treated fabric was then wrapped and fixed on the protected skin using double-sided Scotch® tape at the beginning of the second phalanx. Chemical treatments were randomized. Ten ticks were placed at the tip of the untreated forefinger and left to climb for 10 min. At the end of the experiment, ticks were described as repelled if they fell from the finger or remained on the tip of the first phalanx. Nymphs were described as non-repelled if they stayed on the treated fabric or if they crossed the treated portion of the finger. For each tick group used, the same ticks used in the treated groups were first tested in control treatments to ensure the individual attempted to climb and did not display non-ambulatory behavior that would be viewed as repellency in the treatment group. Each chemical and oil was tested at a concentration of 10 μg/cm^2^ dissolved in acetone. Between each tick group, hands were washed with unscented soap and de-ionized water to avoid the presence of the previous chemical tested. Three to five replicates were performed for each compound studied with each replicate containing 10 individual ticks.

### Statistical analysis

Statistical analyses were performed using GraphPad Prism 9 (GraphPad Software, Inc., San Diego, CA, USA). The percentage of presence in the control zone (% in Ctl zone) for the contact and spatial assay was modified using the following formula, $100-(\left(1-\frac{\% in \,Ctl \,zone}{100}\right)\times 200)$, to obtain comparable percentage of repellency among the three different bioassays. The percentages of repellency for all assays were compared between the mean of control groups (only in presence of acetone) and tested groups (with one side treated with a compound) using ordinary two-way ANOVA and uncorrected Fisher’s LSD test with a single pooled variance for spatial assay. Kruskal-Wallis tests with uncorrected Dunn’s test were performed for the contact repellent bioassay. Repellency data for all remaining analyses were corrected against pre-trial data using Abbott’s corrected mortality formula [40]. The RC_50_ of TCA was obtained by non-linear regression to a four-parameter logistic equation using GraphPad Prism software. Multiple paired t-test was used to compare control and tested repellency obtained for the fingertip assay. A correlation matrix on the percentage of repellency from the three different bioassays for all chemicals and natural products at 10 μg/cm^2^ was calculated using the non-parametric Spearman correlation coefficient.

## Results

### Contact repellency

**Fig. 2:**
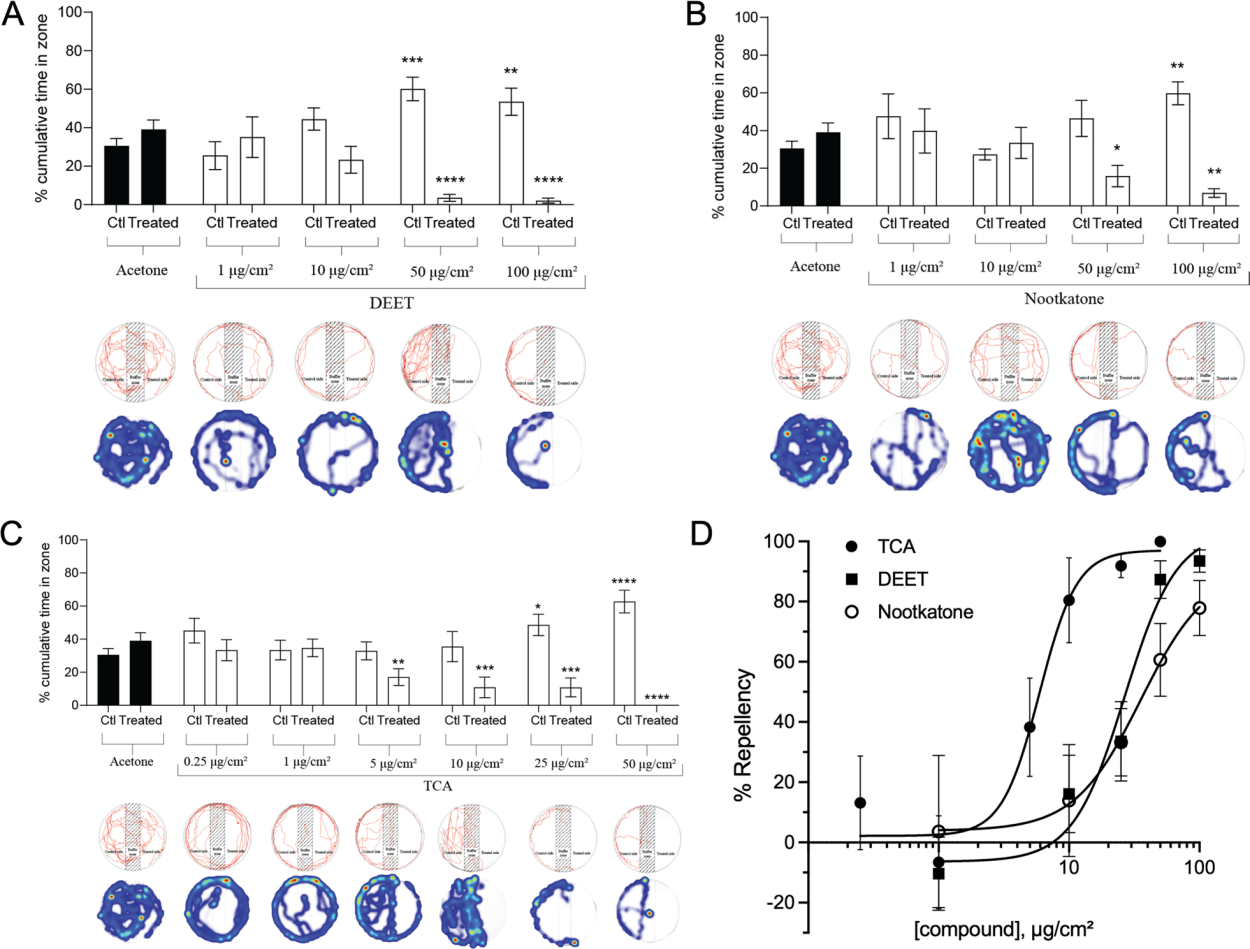
Contact repellency of *Amblyomma americanum* nymphs. DEET (**A**), nootkatone (**B**) and TCA **(C)** contact repellency compared to acetone control is shown based on total time spent in the untreated control (ctl) zones compared to treated zones. Bars represent mean (6 replicates, 10 ticks per replicate), and error bars represent SEM. Asterisks represent statistical significance between treated and control repellency within the same concentration where *P < 0.05, **P < 0.01, ***P < 0.001 and ****P < 0.0001 as determined by an unpaired Student's t-test. Below each concentration, representative movement trackers are shown as a line map (top) and heat map (bottom) generated by Ethovision software showing movements of a single nymphal tick over the course of 60 s for each compound and each concentration. The heat map indicates time spent in the location with blue equaling less time and red equaling more time on average. **D** Concentration-response curves for TCA-, DEET- and nootkatone-mediated repellency. Each data point represents mean (*n* = 10 ticks per concentration) repellency, and error bars represent SD

Acetone was shown to have no repellent or attractive activity to ticks (Table 1). The percent time spent in the control versus treated areas of the arena and representative Ethovision behavior traces for DEET, nootkatone and TCA are shown in Fig. 2A–C, respectively. TCA was the most potent repellent studied in the contact assay with an RC_50_ value of 5.9 μg/cm^2^ (95% CI 2.5–8.5 μg/cm^2^, Hillslope: 3.0, r^2^: 0.64), which was approximately five- and seven-fold more repellent than DEET (RC_50_: 26.7 μg/cm^2^, 95% CI 14–40 μg/cm^2^, Hillslope: 2.1, *R*^2^: 0.79) and nootkatone (RC_50_: 35.4 μg/cm^2^, 95% CI 12–52 μg/cm^2^, Hillslope: 1.4, *R*^2^: 0.49), respectively (Fig. 2D). The RC_50_ of TCA was significantly (*P* < 0.01) lower than the RC_50_ for DEET and nootkatone (Fig. 2D).

**Table 1:** Contact repellency of *Amblyomma americanum* nymphs after exposure to essential oils

Treatment	μg/cm^2^	Repellency (± SEM) %
Acetone	0	− 10.7 (± 10.9)
Geranium	10	4.0 (± 20.0) Aa
	50	89.5 (± 7.2) Ba
Oregano	10	− 13.2 (± 22.5) Aa
	50	86.5 (± 6.6) Ba
Cedarwood	10	30.7 (± 19.0) Ab
	50	83.7 (± 7.6) Bb
Thyme	10	40.7 (± 19.2) Ab
	50	81.6 (± 8.2) Bb
Clove	10	49.1 (± 22.0) Ab
	50	91.3 (± 4.8) Ba
Juniper berry	10	78.0 (± 13.5) Bd
	50	44.8 (± 26.3) Ac
Amyris	10	30.8 (± 23.9) Ab
	50	78.9 (± 11.8) Bc
Cassia	10	27.3 (± 24.2) Ab
	50	1.9 (± 25.3) Ad
Citronella	10	92.4 (± 4.3) Bd
	50	61.7 (± 5.6) Bc
Peppermint	10	6.2 (± 30.0) Aa
	50	30.3 (± 33.5) Ac
Patchouli	10	43.1 (± 35.2) Ab
	50	62.8 (± 22.3) Bc
Fennel	10	58.3 (± 13.3) Bc
	50	60.2 (± 22.4) Bc
Lemon	10	− 20.3 (± 36.8) Aa
	50	67.5 (± 19.3) Bc
Dill	10	49.2 (± 19.1) Ab
	50	80.1 (± 5.6) Bc
Black pepper	10	45.8 (± 36.3) Ab
	50	63.2 (± 17.5) Bc
Lavender	10	0.46 (± 31) Aa
	50	79.4 (± 13.1) Bc

Percentage of nymphs repelled is presented as mean (6 replicates, 10 ticks per replicate) ± SEM for each treatment and both concentrations. Statistical significance is denoted by letters where uppercase letters represent statistical significance at *P* < 0.05 compared to solvent control and lowercase letters represent compared to other oils at the same concentration. Groups not labeled by the same uppercase or lowercase letter represent statistical significance at *P* < 0.05 as determined by an unpaired t-test (comparison to solvent control) or one-way ANOVA with Tukeys posttest (comparison of oils)

At 10 μg/cm^2^, juniper berry and citronella significantly repelled ticks from the treated surface as the percentage of repellency was 8.3- and 9.6-fold more than for control treatments, which was a statistically (*P* < 0.01) significant increase (Table 1). Interestingly, juniper berry and citronella were the only two essential oils that exhibited > 75% repellency (Table 1) at 10 μg/cm^2^. Rank order of potency at 10 μg/cm^2^ was shown to be TCA = citronella = juniper berry > fennel > dill = clove = black pepper = patchouli = thyme = DEET = amyris = cedarwood = cassia > peppermint = geranium = lavender = nootkatone = oregano = lemon (Table 1, Fig. 2D).

Repellent activity at 50 μg/cm^2^ was significantly (*P* < 0.01) increased for nearly all essential oils tested compared to control and 10 μg/cm^2^ (Table 1). Rank order of potency at 50 μg/cm^2^ was shown to be TCA = clove = geranium = DEET = oregano = cedarwood > thyme > dill = lavender = amyris = lemon = black pepper = patchouli = citronella = fennel = nootkatone > juniper berry = peppermint > cassia (Table 1). For instance, geranium was 9.4-fold more potent, oregano was 9.1-fold more potent, cedarwood was 8.8-fold more potent, thyme was 8.6-fold more potent, clove was 9.5-fold more potent, amyris was 8.4-fold more potent, patchouli was 6.9-fold more potent, fennel was 6.6-fold more potent, lemon was 7.3-fold more potent, dill was 8.5-fold more potent, black pepper was 6.9-fold more potent and lavender was 8.4-fold more potent compared to control. Interestingly, a significant reduction in potency for citronella and juniper berry was observed at 50 μg/cm^2^ compared to 10 μg/cm^2^ (Table 1).

### Spatial bioassay

TCA was not an effective spatial repellent at 1 μg/cm^2^ but was spatially active at 10 μg/cm^2^ with significant (*P* < 0.05) reduction of tick presence in the treated area at 30 min, 1 h and 2 h exposure time points compared to control (Table 2). An increase in TCA-mediated repellency was observed at 50 μg/cm^2^ or all recorded time points compared to control and 10 μg/cm^2^. A 4.7-, 6.8-, 8.1- and 9.9-fold reduction of presence in the treated side of the tube was observed at 15 min, 30 min, 1 h and 2 h, respectively, after exposure to 50 μg/cm^2^ TCA, which were all statistically significant (*P* < 0.01) compared to control (Table 2). Interestingly, 50 μg/cm^2^ TCA repelled 90 ± 10% of ticks at an exposure time of 30 min, which was 1.7- and 6.9-fold more effective than DEET and nootkatone, respectively; these were statistically significant (*P* < 0.05) differences in repellency compared to control.

**Table 2:** Spatial repellency of *Amblyomma amblyomma* nymphs over time after exposure to 16 essential oils, DEET, nootkatone and TCA

		15 min	30 min	1 h	2 h
Treatment	μg/cm^2^	Repellency (± SEM) %	Repellency (± SEM) %	Repellency (± SEM) %	Repellency (± SEM) %
Acetone	0	− 12.2 (± 9.04)	− 15.7 (± 9.82)	− 11.3 (± 11.7)	− 9.57 (± 12.6)
DEET	1	20 (± 21.6)	5 (± 20.6)	25 (± 22.2)	35 (± 22.2)
	10	8 (± 17.4)	24 (± 19.4)	4 (± 24.8)	24 (± 31.87)
	50	20 (± 17.1)	53.3 (± 20.4)	83.3 (± 8)	86.7 (± 6.7)
Nootkatone	1	− 15 (± 31)	− 20 (± 21.6)	− 20 (± 24.5)	20 (± 31.6)
	10	16 (± 24.8)	32 (± 16.3)	12 (± 24.2)	40 (± 24.5)
	50	0 (± 18.6)	13.3 (± 22.3)	23.3 (± 13.1)	66.7 (± 18.4)
TCA	1	− 25 (± 17.1)	− 5 (± 18.9)	5 (± 26.3)	0 (± 29.4)
	10	25 (± 33)	50 (± 19.2)	30 (± 12.9)	65 (± 12.6)
	50	45 (± 17.1)	90 (± 10)	80 (± 14.1)	95 (± 5)
Geranium	1	16.4 (± 34.8)	9.7 (± 28.9)	9.1 (± 24.8)	14.6 (± 30)
	10	50 (± 33.2)	55 (± 28.7)	55 (± 33)	60 (± 18.3)
Oregano	1	− 20 (± 40)	− 20 (± 30.6)	− 13.3 (± 24)	− 26.7 (± 24)
	10	26.7 (± 40.6)	13.3 (± 43.7)	66.7 (± 17.6)	73.3 (± 17.6)
Cedarwood	1	− 33.3 (± 17.6)	− 53.3 (± 6.7)	− 26.7 (± 33.3)	− 20 (± 30.6)
	10	53.3 (± 17.6)	20 (± 11.6)	20 (± 23.1)	20 (± 11.6)
Thyme	1	26.7 (± 13.3)	46.7 (± 29.1)	73.3 (± 17.6)	66.7 (± 24)
	10	100	93.3 (± 6.7)	93.3 (± 6.7)	86.7 (± 13.3)
Clove	1	− 4.1 (± 19.1)	− 5 (± 49.9)	− 5 (± 45.7)	− 15 (± 46.5)
	10	60 (± 11.6)	66.7 (± 6.7)	80	86.7 (± 6.7)
Juniper berry	1	5 (± 26.3)	10 (± 25.2)	15 (± 25)	15 (± 28.7)
	10	53.3 (± 6.7)	60 (± 11.6)	53.3 (± 13.3)	53.3 (± 24)
Amyris	1	10 (± 48)	10 (± 40.4)	40 (± 24.5)	5 (± 17.1)
	10	− 15 (± 27.5)	− 5 (± 20.6)	0 (± 18.3)	20 (± 16.3)
Cassia	1	20 (± 29.4)	10 (± 33.2)	25 (± 41.1)	25 (± 35)
	10	60 (± 23.1)	93.3 (± 6.7)	93.3 (± 6.7)	100
Citronella	1	55 (± 26.3)	60 (± 21.6)	70 (± 23.8)	75 (± 25)
	10	− 13.3 (± 17.6)	− 13.3 (± 13.3)	0 (± 20)	− 26.7 (± 6.7)
Peppermint	1	3.2 (± 30.4)	8.2 (± 27.2)	12.3 (± 33)	12.3 (± 33)
	10	− 20 (± 31.6)	− 15 (± 29.9)	− 5 (± 33)	− 10 (± 46.6)
Patchouli	1	60 (± 18.3)	30 (± 25.2)	50 (± 30)	65 (± 23.6)
	10	20 (± 11.6)	20 (± 20)	33.3 (± 29.1)	40 (± 20)
Fennel	1	5 (± 27.5)	15 (± 29.9)	35 (± 27.5)	30 (± 31.1)
	10	15 (± 33.0)	25 (± 29.9)	0 (± 27.1)	− 5 (± 35.9)
Lemon	1	46.7 (± 43.7)	33.3 (± 46.7)	53.3 (± 26.7)	60.0 (± 30.6)
	10	10.0 (± 10.0)	− 5 (± 15.0)	25.0 (± 15.0)	20.0 (± 18.3)
Dill	1	13.3 (± 29.1)	13.3 (± 37.1)	0 (± 40.0)	6.7 (± 33.3)
	10	20.0 (± 24.5)	15.0 (± 23.6)	45.0 (± 22.2)	55.0 (± 22.2)
Black pepper	1	15 (± 26.3)	15 (± 27.5)	35 (± 32.0)	25 (± 33.0)
	10	35 (± 15.0)	35 (± 20.6)	80 (± 20.0)	80 (± 14.1)
Lavender	1	30 (± 23.8)	40 (± 24.5)	50 (± 20.8)	60 (± 14.1)
	10	25 (± 35.0)	35 (± 29.9)	55 (± 12.6)	45 (± 23.6)

The percentage of nymphs repelled over time is represented as mean (6 replicates) ± SEM at 15 min, 30 min, 1 h and 2 h for each treatment at all concentrations tested

At 50 μg/cm^2^, DEET was active as a spatial repellent at all time points tested. A 4.4-, 8.4- and 10.1-fold reduction of presence in the treated side of the tube was observed at 30 min, 1 h and 2 h, respectively, from 50 μg/cm^2^ DEET, which were all statistically significant (*P* < 0.01 at 30 min, *P* < 0.001 at 1 h and 2 h) compared to control (Table 2). At 100 μg/cm^2^ DEET, tick presence in the treated side of the tubes was reduced by 3.8-fold at 30 min (*P* = 0.012, Table 2), 7-fold at 1 h (*P* < 0.001) and 11-fold at 2 h (*P* < 0.001) compared to the control groups (Table 2).

Nootkatone, which is a natural compound and is registered by the EPA as a tick repellent [41], was less active than DEET and TCA in the spatial assay with no significant repellency at 30 min or 1 h when tested at 50 μg/cm^2^ but significantly (*P* < 0.01) repelled ticks at 2 h post-exposure (Table 2). Nootkatone was more effective at 100 μg/cm^2^ where ticks were significantly repelled at 15 min (*P* < 0.01), 30 min (*P* < 0.01), 1 h (*P* < 0.001) and 2 h (*P* < 0.001).

Of the essential oils tested at 1 μg/cm^2^, patchouli and citronella were the two most repellent molecules in the spatial repellent assay at the earliest time point of 15 min with 60 ± 18% and 55 ± 26% of ticks repelled (Table 2). The rapid repellent activity of pachouli and citronella oils is relevant because no other oil significantly repelled ticks at this early time point with 1 μg/cm^2^. Yet, it is important to note that patchouli did not significantly repel ticks at 30 min or 1 h despite the high activity at 15 min (Table 1). Contrary to pachouli, 1 μg/cm^2^ citronella oil was an effective spatial repellent at all time points tested with a decrease of ticks in the treated zone by 5.5-, 4.8-, 7.1- and 8.8-fold at 15 min, 30 min, 1 h and 2 h post-exposure, which were all significantly (*P* < 0.05) reduced compared to control. Although less repellent than pachouli and citronella oils, exposures to volatiles from 1 μg/cm^2^ thyme and lavender oils were strong spatial repellents at 1 and 2 h post-exposure and were significantly (*P* < 0.05) different from control treatments (Table 2). All other oils at 1 μg/cm^2^ were not effective spatial repellents at any time point tested.

At 10 μg/cm^2^, amyris, citronella, peppermint, patchouli, fennel and lemon oils did not demonstrate any spatial repellent activity (*P* > 0.05) against *Am. americanum* nymphs (Table 2). Thyme and cassia were the two most active spatial repellents studied at 10 μg/cm^2^ with rapid rates of repellency that were sustained throughout the study period. Thyme was the most potent and effective spatial repellent when tested at 10 μg/cm^2^ with 100% repellency at 15 min exposure, which was the earliest time point tested, and repellency was not reduced at later time points (Table 2). Cassia was also an extremely effective spatial repellent at 10 μg/cm^2^ with 60 ± 17.6% repellency at 15 min and > 90% repellency at all subsequent time points (Table 2). Clove and cedarwood were not significantly different (*P* > 0.05) from each other at 15-min exposure and were the second most potent oils tested with 60 ± 11.6% and 53.3 ± 17.6% tick repellency. The spatial repellent activity of clove significantly increased (*P* < 0.05) at 1 h and 2 h time points compared to 15 min exposure, but interestingly, potency of cedarwood was significantly (*P* < 0.05) reduced at 30 min, 1 h and 2 h time points compared to 15 min (Table 2). Black pepper was slower to induce repellency compared to clove or cedarwood but was an effective spatial repellent at 10 μg/cm^2^ after 1- and 2-h exposure times with 80 ± 20% and 80 ± 14% repellency, respectively.

### Fingertip repellent bioassay

**Fig. 3:**
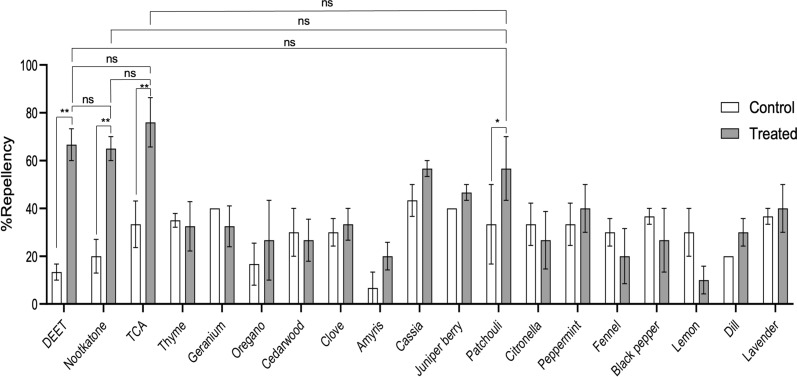
Fingertip repellency bioassay of *Amblyomma americanum* nymphs. Repellent potency of 16 essential oils, TCA, nootkatone and DEET in an in vivo repellent assay performed on a human volunteer (UF IRB: IRB202301534). Bars represent mean (*n* = 5 ticks) percent repellency, and error bars represent SD. Asterisks represent statistical significance between treated and control repellency within the same compound where *P < 0.05 and **P < 0.01 as determined by a multiple paired t-test. Non-significance is denoted by “ns” and is *P* > 0.05

No significant difference in repellency was observed among TCA, DEET and nootkatone at the tested concentration of 10 μg/cm^2^, but all three compounds resulted in significant (*P* < 0.01) repellency compared to solvent control treatments (Fig. 3). Surprisingly, patchouli oil was the only essential oil tested that led to a significant (*P* < 0.05) repellency of ticks with a reduction of 1.7-fold compared to solvent control (Fig. 3). The rank order of repellency in the fingertip assay with *Am. americanum* nymphs is TCA = nootkatone = DEET > patchouli > cassia > juniper berry = peppermint = lavender = clove = thyme = geranium = oregano = dill = cedarwood = citronella = black pepper = amyris = fennel = lemon.

### Correlation among the three repellent bioassays

**Fig. 4:**
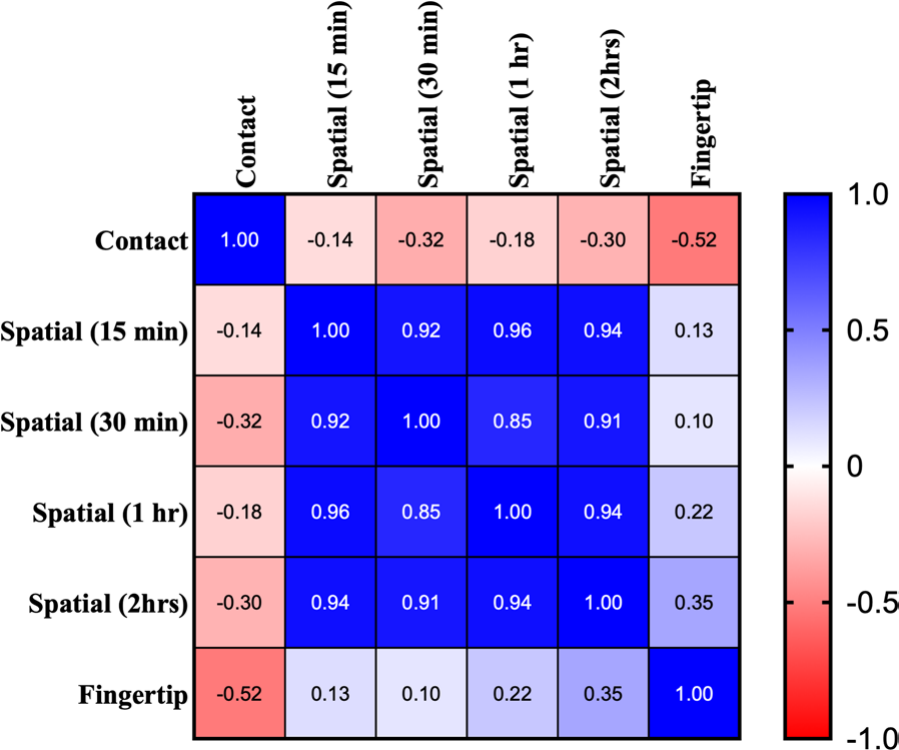
Correlation matrix of repellency among contact, spatial (for all time recorded) and fingertip bioassays. The correlation matrix represents the correlation from the percent repellency for all the bioassays at 10 μg/cm^2^ for each compound. Compounds included in the analysis were DEET, nootkatone, TCA, citronella, patchouli, thyme, clove, cassia, juniper berry and fennel. Compounds that were < 50% repellent in all assays were excluded from analysis to eliminate the positive correlation of poor tick repellents. The value indicated for each comparison represents the correlation indicator that is illustrated by the scale on the right side of the matrix where + 1.0 is complete positive correlation and − 1.0 is a complete negative correlation

We aimed to test the correlation of repellency among the three assays through generation of a correlation matrix (Fig. 4). The inputs into this matrix were the percent repellency at 10 μg/cm^2^ of TCA, DEET (synthetic standard), nootkatone (natural standard) and the top performing essential oils in each assay (patchouli, citronella, fennel, thyme, clove and cassis). A negative correlation was identified between the fingertip and contact assay, indicating the repellents that were active in one assay were inactive in the other. A weak, positive correlation was observed between latent time points (e.g. 60 min) of the spatial and fingertip assay; similarly, a weak negative correlation was observed between any time point of the spatial assay and the contact assay (Fig. 4). As expected, the different time points within the spatial assay were shown to be positively correlated (0.85 < r < 0.96, Fig. 4).

## Discussion

Although significant scientific progress has been achieved in the fields of tick genomics, secreted salivary proteins and vaccine technology, these advancements have translated poorly into successful commercialization of therapeutics to reduce morbidity and mortality stemming from tick-vectored pathogens [42–50]. While the development of novel acaricides and vaccine technologies remain important endeavors, the ability to repel tick vectors from human hosts represents a cheap and viable option to reduce tick-borne pathogen transmission to humans. However, innovation in the field of arthropod repellents has been relatively low and continues to rely heavily on DEET, which some are hesitant to use even though it has been accepted as safe when used correctly [9, 10, 51], IR3535 or picaridin. Considering this, recent efforts have been made to develop novel repellents that can be integrated into the current rotation of insecticides and personal protectants for protection from arthropod-vectored pathogens [52–55].

A challenge to the development of novel chemical insecticides or repellents is the time required to move from “bench to field” for new chemistry, which hinders the ability to address the ongoing surge of tick-borne diseases. One of the primary routes for rapid commercialization of pesticides is to develop compounds of natural origin (i.e. biopesticides), which are significantly cheaper and faster to commercialize than synthetic pesticides [56, 57]. To this point, nootkatone is a component of grapefruit oil that has repellent and toxic properties against ticks [58, 59] and has recently been registered by the EPA for arthropod control after approximately 3 years of development [41, 60, 61]. This relatively short time from development to EPA registration for nootkatone highlights the rapid advancement through regulatory checks for biopesticides and the potential for natural products to become commercialized products directed to tick control. Thus, the goal of this study was two-fold: (i) to test the tick repellency of natural product extracts or natural compounds that are known to have repellent properties against mosquitoes and (ii) to assess the correlation of three distinct repellent assays that can inform downstream studies for appropriate assays that translate to field repellency and can inform assays used for EPA registration.

The acid moieties of pyrethroids and natural pyrethrin I have significant repellent activity against mosquitoes [38, 53, 54]. More specifically, 1R-trans-permethrinic acid (TFA) and 1R-trans-chrysanthemic acid (TCA), which are derived from hydrolysis of permethrin and natural pyrethrin I, respectively, provided significant repellency to mosquitoes and prevented mosquito bites on human arms [38, 53, 54]. The high repellent activity of TCA to mosquitoes is significant for preventing tick bites because it is a natural compound (i.e. natural pyrethrin I) that can meet the need for an effective tick repellent with potential for rapid advancement through the regulatory pipeline. Therefore, we tested the repellent activity of TCA against *Am. americanum* in three distinct bioassays compared to DEET and nootkatone. TCA was significantly more repellent to *Am. americanum* nymphs than DEET, nootkatone and essential oils in the contact and spatial repellent assays (Fig. 2, Tables 1, 2) but was equal in activity to DEET and nootkatone in the fingertip assay (Fig. 3). These data indicate that TCA represents a new naturally occurring active compound that can control tick populations or prevent ticks from biting humans. Importantly, TCA has a positive mammalian safety profile with a mouse oral LD_50_ value of 364 mg/kg (95% CI 200–600 mg/kg), which is not significantly different from transfluthrin [37, 38, 53], which is a commercialized spatial repellent for ticks and mosquitoes and does not present risk of concern to human health when used according to label instructions [62]. Although TCA was shown to repel ticks at a distance remote from the source, it is necessary to define the repellency of TCA in field conditions as host acquisition is often due to hosts contacting the tick rather than ticks moving to a host. Spatial repellency is likely less relevant for most tick species than for mosquitoes, yet the high spatial repellency of TCA may provide significant benefit to certain tick species, such as the Brown dog tick (*Rhipicephalus sanguineus*), which are associated with dog kennels and places where dogs reside. The translation of TCA activity to *R. sanguineus* is also significant as TCA was shown to be equally repellent to pyrethroid-resistant mosquitoes carrying kdr mutations compared to pyrethroid-susceptible mosquitoes [38], which is relevant because of multiple reports of high pyrethroid resistance in this species [63, 64].

Previous studies have documented several oils extracted from plants or naturally derived compounds as active tick repellents [19, 24, 65–67]. This finding continues to support the notion that natural compounds can be used as an effective tool to reduce tick bites. Interestingly, using a vertical filter paper assay, some natural compounds tested have been described as less effective than DEET against *Am. americanum* nymphs [22, 24], yet oil of lemon eucalyptus was more active than DEET, picaridin and IR3535 when tested using the tick carousel assay [28]. Contrarily, repellent potency of DEET, peppermint oil and rosemary oil against *I. scapularis* did not differ significantly between in vitro (jar and petri dish) and in vivo assays (fingertip and forearm) [30]. Thus, the second goal of this study was to assess how the repellent potencies of 16 essential oils, the naturally derived pyrethrin acid, TCA, nootkatone and DEET vary based on two in vitro assays and one in vivo assay. High variability was noted across the three bioassays with multiple essential oils being better than DEET and nootkatone at 10 μg/cm^2^ and 50 μg/cm^2^ in the contact and spatial assays (Tables 1 and 2), but they were less active than DEET in the fingertip assay (Fig. 3). Similarly, thyme was shown to be the most repellent oil tested in the spatial assay (Table 2) but was 6.7-fold less active than DEET in a vertical climbing assay [24]. These data are summarized in Fig. 4, which shows the repellent activity identified in spatial or contact assays is negatively correlated with repellent efficacy determined in the fingertip assay. The negative correlation of repellency based on assay type was somewhat surprising as we anticipated that highly volatile oils would be effective repellents in the contact and spatial bioassays. However, repellent activity was negatively correlated between these two assays, which suggests volatility is not the only metric for predicting efficacy in these assays. Similarly, we anticipated that repellent activity would be correlated between the contact and fingertip bioassays because both assays incorporate tick contact with a substrate. Yet, repellent activity was negatively correlated between these two assays, which suggests a series of currently undefined physiochemical parameters is important for predicting biological activity in these assays.

Correlation analysis was performed to relate the ability of each repellent assay to inform the performance of the others. It was observed that performance in the contact assay was negatively correlated (albeit weakly) with performance in the spatial repellency assay. This was expected as volatility is requisite for spatial repellency; however, increasing volatility leads to decreased contact repellency (primarily by decreasing the duration of effect). This is well documented for natural products, as many are not long-lasting repellents because of their relatively high volatility profiles [68]. Moreover, Paluch et al. [69] demonstrated that vapor pressure was negatively correlated with contact repellency and early stage spatial repellency. The authors postulate that lower vapor pressure natural products likely possess higher concentrations on treated filter papers for longer, which is particularly important for contact repellency. Unexpectedly, there was a negative correlation between the fingertip and contact repellency assay. This could be due to a variety of factors, e.g., (i) host volatiles in the fingertip assay fundamentally change the outcomes compared to both the contact assay (physical interactions with the repellents or modification of the tick’s physiology compared to the in vitro assays), (ii) the fabric used in the human fingertip assay causes treatments to behave differently than when applied to filter papers in the contact assay, (iii) heat of the finger changes the physical properties of the compounds screened and/or physiology of the ticks or (iv) volatile (spatial) repellents are more effective in this assay system. Notably, a weak, positive correlation was observed between the fingertip and spatial repellency assay. This finding could indicate that natural product odorants that are volatile might be more appropriate for development of human skin or fabric repellents aimed at controlling ticks than non-volatile (contact) repellents. However, more work is needed to understand this trend further and to document it beyond natural product repellents.

## Conclusions

In ﻿conclusion, TCA repelled *Am. americanum* nymphs equally to or better than DEET and nootkatone in the three assays we used to quantify repellency, which, when combined with the high mammalian safety profile and high activity to pyrethroid-resistant insects [38, 53], suggests it represents a candidate tick repellent to protect humans from tick bites. Importantly, the developmental pipeline for TCA is likely to be less than a synthetic repellent as it is derived from natural pyrethrin I. The differences in repellent potency based on the assay type (Fig. 4) suggest that the ability to discover active tick repellents suitable for development is more complicated than for other arthropod species; furthermore, the field delivery mechanism must be considered early in development to ensure translation to field efficacy. Future work should aim to define the membrane proteins mediating reception and transduction in chemosensory neurons of ticks, which have been well characterized in mosquitoes [70]. This will aid in defining the mode of repellency for TCA and development of novel mechanisms for tick repellents.

### Supplementary Information

**Additional file 1: Table S1.** GC/MS analysis of plant oil constituents.

## Data Availability

The data that support the findings of this study are available from the corresponding author upon reasonable request.

## Abbreviations

DEET: N,N-diethyl-*m*-toluamide
RC_50_: Effective concentration to repel 50% of the ticks
PMD: *p*-Menthane-3,8-diol
TCA: 1R-trans-chrysanthemic acid
