# Supplementary material for: Repellent activity of essential oils to the Lone Star tick, Amblyomma americanum
Source: Parasit Vectors. 2024 May 6;17:202. doi: 10.1186/s13071-024-06246-0 (PMC11073969; doi:10.1186/s13071-024-06246-0)
Supplement: Supplementary file 1 — Additional file 1: Table S1. GC/MS analysis of plant oil constituents. [file 13071_2024_6246_MOESM1_ESM.docx]

**Additional file Table 1. GC/MS Analysis of Plant Oil Constituents**

| fennel | | Component | RT | Area (counts*min) | Relative Height |
| --- | --- | --- | --- | --- | --- |
|  | (1R)-2,6,6-Trimethylbicyclo[3.1.1]hept-2-ene | | 5.8 | 173147217 | 3.37 |
|  | Bicyclo[3.1.1]hept-2-ene, 3,6,6-trimethyl- | | 5.93 | 10428582 | 0.20 |
|  | Hydroperoxide, 1-ethylbutyl | | 6.11 | 30098030 | 0.59 |
|  | Hydroperoxide, 1-methylpentyl | | 6.42 | 27839468 | 0.54 |
|  | 2-Pentene, 2,4-dimethyl- | | 6.84 | 137546101 | 2.68 |
|  | Bicyclo[3.1.1]heptane, 6,6-dimethyl-2-methylene-, (1S)- | | 7.58 | 24576518 | 0.48 |
|  | 2-Pentene, 2,4-dimethyl- | | 7.66 | 10348743 | 0.20 |
|  | 2-Pentene, 2,4-dimethyl- | | 7.75 | 12546112 | 0.24 |
|  | Bicyclo[3.1.0]hex-2-ene, 2-methyl-5-(1-methylethyl)- | | 8.13 | 13000566 | 0.25 |
|  | 3-Carene | | 8.21 | 17352052 | 0.34 |
|  | o-Cymene | | 8.8 | 14046627 | 0.27 |
|  | D-Limonene | | 8.97 | 113571194 | 2.21 |
|  | D-sylvestrene | | 9.01 | 254819628 | 4.96 |
|  | D-Limonene | | 9.08 | 529311187 | 10.30 |
|  | L-Fenchone | | 11.24 | 114879903 | 2.24 |
|  | L-Fenchone | | 11.29 | 154555632 | 3.01 |
|  | Linalool | | 11.83 | 33843142 | 0.66 |
|  | 3-Cyclohexen-1-ol, 4-methyl-1-(1-methylethyl)-, (R)- | | 15.15 | 11632837 | 0.23 |
|  | Estragole | | 15.97 | 122557946 | 2.39 |
|  | 5-(2H-1,3-Benzodioxol-5-ylmethyl)-3-(3-methoxyphenyl)-4,5-dihydro-1,2-oxazole | | 18.31 | 43536926 | 0.85 |
|  | 5-(2H-1,3-Benzodioxol-5-ylmethyl)-3-(3-methoxyphenyl)-4,5-dihydro-1,2-oxazole | | 18.35 | 32530399 | 0.63 |
|  | Anethole | | 20 | 3163796417 | 61.58 |
|  | Formic acid, 2-isopropylphenyl ester | | 23.68 | 17633880 | 0.34 |
|  | 2-Propanone, 1-(4-methoxyphenyl)- | | 23.77 | 16610941 | 0.32 |
|  | cis-a-Bergamotene | | 25.98 | 16806751 | 0.33 |
|  | 1-(3-Methyl-2-butenoxy)-4-(1-propenyl)benzene | | 35.4 | 40331432 | 0.79 |
|  |  | |  | 5137348231 |  |
|  |  | |  |  | 100.00 |

| lavender | Component | RT | Area (counts*min) | Relative Height |
| --- | --- | --- | --- | --- |
|  | Bicyclo[3.1.0]hex-2-ene, 4-methyl-1-(1-methylethyl)- | 5.63 | 4836430 | 0.08 |
|  | 3-Carene | 5.83 | 13042452 | 0.22 |
|  | Ethanone, 1-(3-ethyloxiranyl)- | 6.17 | 27854159 | 0.47 |
|  | Camphene | 6.3 | 5630948 | 0.10 |
|  | Sulfurous acid, butyl isohexyl ester | 6.47 | 29020040 | 0.49 |
|  | 2-Pentene, 2,4-dimethyl- | 6.9 | 127370015 | 2.16 |
|  | 1-Octen-3-ol | 7.3 | 9614386 | 0.16 |
|  | 3-Octanone | 7.47 | 62724314 | 1.06 |
|  | Bicyclo[3.1.1]heptane, 6,6-dimethyl-2-methylene-, (1S)- | 7.63 | 42986187 | 0.73 |
|  | (-)-Isopinocampheol, trifluoroacetate | 7.72 | 21734178 | 0.37 |
|  | Linalool | 7.85 | 24157576 | 0.41 |
|  | 3-Octanol | 7.88 | 19577286 | 0.33 |
|  | Acetic acid, hexyl ester | 8.44 | 35103946 | 0.60 |
|  | o-Cymene | 8.83 | 25750464 | 0.44 |
|  | Cyclohexene, 1-methyl-5-(1-methylethenyl)-, (R)- | 9.02 | 9764716 | 0.17 |
|  | Eucalyptol | 9.1 | 71412437 | 1.21 |
|  | Bicyclo[3.1.1]hept-2-ene, 3,6,6-trimethyl- | 9.35 | 165706789 | 2.81 |
|  | ß-Ocimene | 9.8 | 92743705 | 1.57 |
|  | 2-Furanmethanol, 5-ethenyltetrahydro-a,a,5-trimethyl-, cis- | 10.63 | 19483153 | 0.33 |
|  | 3-Oxatricyclo[4.1.1.0(2,4)]octane, 2,7,7-trimethyl- | 10.83 | 13267142 | 0.22 |
|  | trans-Linalool oxide (furanoid) | 11.29 | 13452029 | 0.23 |
|  | Linalool | 12.01 | 2379542905 | 40.34 |
|  | 1-Octen-3-yl-acetate | 12.33 | 62188789 | 1.05 |
|  | Bicyclo[3.1.0]hex-2-ene, 4,4,6,6-tetramethyl- | 13.09 | 21334834 | 0.36 |
|  | Bicyclo[2.2.1]heptan-2-one, 1,7,7-trimethyl-, (1S)- | 13.69 | 13199410 | 0.22 |
|  | 4-Hexen-1-ol, 5-methyl-2-(1-methylethenyl)- | 14.62 | 52069055 | 0.88 |
|  | endo-Borneol | 14.81 | 39847720 | 0.68 |
|  | 3-Cyclohexen-1-ol, 4-methyl-1-(1-methylethyl)-, (R)- | 15.24 | 377433685 | 6.40 |
|  | 3-Cyclohexene-1-methanol, a,a,4-trimethyl-, (R)- | 15.87 | 96999716 | 1.64 |
|  | Ethanol, 2-(3,3-dimethylcyclohexylidene)-, (Z)- | 17.24 | 12883134 | 0.22 |
|  | Linalyl acetate | 18.49 | 1215079731 | 20.60 |
|  | Bicyclo[2.2.1]heptan-2-ol, 1,7,7-trimethyl-, acetate, (1S-endo)- | 19.72 | 10235888 | 0.17 |
|  | 4-Hexen-1-ol, 5-methyl-2-(1-methylethenyl)-, acetate | 19.89 | 193241059 | 3.28 |
|  | 2,6-Octadien-1-ol, 3,7-dimethyl-, acetate, (Z)- | 23.5 | 58978935 | 1.00 |
|  | (1S,5S)-2-Methyl-5-((R)-6-methylhept-5-en-2-yl)bicyclo[3.1.0]hex-2-ene | 24.18 | 13224082 | 0.22 |
|  | Caryophyllene | 25.32 | 214562792 | 3.64 |
|  | Tricyclo[2.2.1.0(2,6)]heptane, 1,7-dimethyl-7-(4-methyl-3-pentenyl)-, (-)- | 25.37 | 24297457 | 0.41 |
|  | cis-a-Bergamotene | 25.99 | 10800732 | 0.18 |
|  | cis-ß-Farnesene | 26.95 | 190624675 | 3.23 |
|  | (1R,2S,6S,7S,8S)-8-Isopropyl-1-methyl-3-methylenetricyclo[4.4.0.02,7]decane-rel- | 27.78 | 14877316 | 0.25 |
|  | Caryophyllene oxide | 31.64 | 61839095 | 1.05 |
|  |  |  | 5898493362 | 100.00 |

| dill | Component | RT | Area (counts*min) | Relative Height |
| --- | --- | --- | --- | --- |
|  | Hydroperoxide, 1-ethylbutyl | 6.16 | 24040809 | 0.63 |
|  | Sulfurous acid, isohexyl 2-propyl ester | 6.47 | 25500510 | 0.67 |
|  | 2-Pentene, 2,4-dimethyl- | 6.8 | 133598640 | 3.49 |
|  | 2-Cyclohexen-1-ol, 1-methyl-4-(1-methylethenyl)-, trans- | 13.31 | 12665019 | 0.33 |
|  | Dill ether | 15.42 | 112564065 | 2.94 |
|  | cis-Dihydrocarvone | 15.93 | 20472560 | 0.53 |
|  | Bicyclo[3.1.0]hexan-3-ol, 4-methylene-1-(1-methylethyl)-, (1a,3a,5a)- | 16.2 | 109149468 | 2.85 |
|  | 1-Oxaspiro[2.5]octan-4-one, 2,2,6-trimethyl-, trans- | 17.02 | 67757064 | 1.77 |
|  | Carvone | 18.11 | 1654407158 | 43.16 |
|  | Thymoquinone | 18.32 | 8932045 | 0.23 |
|  | 1,7,7-Trimethylbicyclo[2.2.1]hept-5-en-2-one | 19.95 | 29261936 | 0.76 |
|  | 1(2H)-Naphthalenone, octahydro-8a-methyl- | 20.23 | 45650266 | 1.19 |
|  | 3-Methyl-4-isopropylphenol | 20.48 | 46432120 | 1.21 |
|  | 6-Hydroxycarvotanacetone | 20.72 | 29996955 | 0.78 |
|  | Bicyclo(3.1.1)heptane-2,3-diol, 2,6,6-trimethyl- | 21.37 | 439587649 | 11.47 |
|  | (1R,2S,4S,5R,7R)-5-isopropyl-1-methyl-3,8-dioxatricyclo[5.1.0.02,4]octane | 21.75 | 41860735 | 1.09 |
|  | Bicyclo(3.1.1)heptane-2,3-diol, 2,6,6-trimethyl- | 21.86 | 14755703 | 0.38 |
|  | 1,2-Cyclohexanediol, 1-methyl-4-(1-methylethenyl)- | 22.38 | 22603015 | 0.59 |
|  | (6R)-7a-Hydroxy-3,6-dimethyl-5,6,7,7a-tetrahydrobenzofuran-2(4H)-one | 23.13 | 12116123 | 0.32 |
|  | (-)-cis-Isopiperitenol | 23.71 | 10264318 | 0.27 |
|  | 2-Octen-1-ol, 3,7-dimethyl-, isobutyrate, (Z)- | 24.16 | 47395626 | 1.24 |
|  | 2,3-Bornanediol | 24.2 | 48949611 | 1.28 |
|  | 1,4-dihydroxy-p-menth-2-ene | 24.62 | 152989398 | 3.99 |
|  | (4R,5R)-4-hydroxy-5-isopropyl-2-methylcyclohex-2-enone2-enone | 25.06 | 18625027 | 0.49 |
|  | 2-Pentyl-cyclohexane-1,4-diol | 25.33 | 63683905 | 1.66 |
|  | (4R,5R)-4-hydroxy-5-isopropyl-2-methylcyclohex-2-enone2-enone | 25.51 | 111154256 | 2.90 |
|  | 2-Cyclohexen-1-one, 4-hydroxy-3-methyl-6-(1-methylethyl)-, trans- | 25.83 | 94989093 | 2.48 |
|  | 2,6,10,10-Tetramethyl-1-oxaspiro[4.5]decan-6-ol | 28.5 | 110887925 | 2.89 |
|  | 2(3H)-Furanone, dihydro-5,5-dimethyl-4-(3-oxobutyl)- | 28.57 | 21288525 | 0.56 |
|  | trans-Dehydroandrosterone, trifluoroacetate | 38.84 | 18678255 | 0.49 |
|  | Sabinyl linoleate | 48.11 | 12073521 | 0.31 |
|  | Limonen-6-ol, pivalate | 49.02 | 11816894 | 0.31 |
|  | Epoxylathyrol | 50.02 | 20993817 | 0.55 |
|  | Limonen-6-ol, pivalate | 50.68 | 10541520 | 0.27 |
|  | Propanoic acid, 2-methyl-, 1-methyl-1-(4-methyl-3-cyclohexen-1-yl)ethyl ester | 50.99 | 23550293 | 0.61 |
|  | Limonen-6-ol, pivalate | 51.27 | 37071209 | 0.97 |
|  | Epoxylathyrol | 51.4 | 2808205 | 0.07 |
|  | Fumagillol | 51.63 | 5736108 | 0.15 |
|  | Limonen-6-ol, pivalate | 51.86 | 10812650 | 0.28 |
|  | 4,11-Dimethyl-8-(propan-2-yl)-5,12-dioxatricyclo[9.1.0.04,6]dodecan-7-ol, Ac | 52.09 | 16323454 | 0.43 |
|  | 1-Isobutyl-7,7-dimethyl-octahydro-isobenzofuran-3a-ol | 52.9 | 39262116 | 1.02 |
|  | Limonen-6-ol, pivalate | 53.14 | 22233320 | 0.58 |
|  | 4,11-Dimethyl-8-(propan-2-yl)-5,12-dioxatricyclo[9.1.0.04,6]dodecan-7-ol, Ac | 53.38 | 25937103 | 0.68 |
|  | Limonen-6-ol, pivalate | 54.07 | 15890605 | 0.41 |
|  | Fumagillol | 54.14 | 16721345 | 0.44 |
|  | Limonen-6-ol, pivalate | 55.42 | 11356812 | 0.30 |
|  |  |  | 3833386751 | 100.00 |

| lemon | Compound | RT | Area (counts*min) | Relative Height |
| --- | --- | --- | --- | --- |
|  | (1R)-2,6,6-Trimethylbicyclo[3.1.1]hept-2-ene | 5.85 | 276496854 | 5.55 |
|  | Ethanone, 1-(3-ethyloxiranyl)- | 6.17 | 29895143 | 0.60 |
|  | Camphene | 6.3 | 12520044 | 0.25 |
|  | Sulfurous acid, isohexyl 2-propyl ester | 6.47 | 26099508 | 0.52 |
|  | Cyclopropane, 2-bromo-1,1,3-trimethyl- | 6.79 | 15977453 | 0.32 |
|  | Oxalic acid, cyclohexyl pentyl ester | 6.89 | 98120474 | 1.97 |
|  | Bicyclo[3.1.0]hexane, 4-methylene-1-(1-methylethyl)- | 7.22 | 354484238 | 7.11 |
|  | o-Cymene | 8.86 | 60082363 | 1.21 |
|  | Cyclohexene, 1-methyl-5-(1-methylethenyl)-, (R)- | 9.03 | 235072574 | 4.72 |
|  | D-Limonene | 9.14 | 805166819 | 16.15 |
|  | Cyclohexene, 1-methyl-5-(1-methylethenyl)-, (R)- | 9.22 | 182539644 | 3.66 |
|  | trans-p-Mentha-2,8-dienol | 12.74 | 98041440 | 1.97 |
|  | 1,7,7-Trimethylbicyclo[2.2.1]hept-5-en-2-ol | 12.9 | 11764220 | 0.24 |
|  | 2-Cyclohexen-1-ol, 1-methyl-4-(1-methylethenyl)-, trans- | 13.39 | 140805827 | 2.82 |
|  | Bicyclo[3.1.1]heptan-3-ol, 6,6-dimethyl-2-methylene-, [1S-(1a,3a,5a)]- | 13.48 | 33288961 | 0.67 |
|  | trans-Verbenol | 13.7 | 13395514 | 0.27 |
|  | Sabinone | 14.35 | 12541688 | 0.25 |
|  | 1H-Inden-1-one, 2,3,4,5,6,7-hexahydro- | 14.58 | 10828626 | 0.22 |
|  | trans-Carveol | 15.13 | 35378912 | 0.71 |
|  | trans-p-mentha-1(7),8-dien-2-ol | 15.54 | 34351271 | 0.69 |
|  | Bicyclo[3.1.1]hept-2-ene-2-carboxaldehyde, 6,6-dimethyl- | 15.77 | 16478946 | 0.33 |
|  | Bicyclo[3.1.1]hept-2-ene-2-methanol, 6,6-dimethyl- | 15.88 | 72743898 | 1.46 |
|  | Tricyclo[2.2.1.0(2,6)]heptane-3-methanol, 2,3-dimethyl- | 16.02 | 37895121 | 0.76 |
|  | (-)-cis-Isopiperitenol | 16.07 | 43021235 | 0.86 |
|  | Bicyclo[3.1.1]hept-3-en-2-one, 4,6,6-trimethyl- | 16.28 | 7361148 | 0.15 |
|  | trans-Carveol | 16.91 | 194223735 | 3.90 |
|  | cis-p-mentha-1(7),8-dien-2-ol | 17.36 | 5359727 | 0.11 |
|  | 2-Cyclohexen-1-ol, 2-methyl-5-(1-methylethenyl)-, cis- | 17.53 | 78448668 | 1.57 |
|  | trans-Verbenol | 17.8 | 12621675 | 0.25 |
|  | Carvone | 17.94 | 63644459 | 1.28 |
|  | Carvone | 17.98 | 71638715 | 1.44 |
|  | 2-Cyclohexen-1-one, 3-methyl-6-(1-methylethenyl)-, (S)- | 18.97 | 12842630 | 0.26 |
|  | 2,6-Octadienal, 3,7-dimethyl-, (E)- | 19.07 | 26242155 | 0.53 |
|  | 1,4-dihydroxy-p-menth-2-ene | 19.24 | 18521592 | 0.37 |
|  | 2-Cyclohexen-1-ol, 2-methyl-5-(1-methylethenyl)-, (1S-trans)- | 19.69 | 19170963 | 0.38 |
|  | 7-Oxabicyclo[4.1.0]heptane, 1-methyl-4-(2-methyloxiranyl)- | 20 | 33872018 | 0.68 |
|  | Thymol | 20.14 | 34425976 | 0.69 |
|  | 5-Isopropenyl-2-methyl-7-oxabicyclo[4.1.0]heptan-2-ol | 20.25 | 24659292 | 0.49 |
|  | (S)-(-)-(4-Isopropenyl-1-cyclohexenyl)methanol | 20.37 | 14096005 | 0.28 |
|  | (3R,6R)-3-Hydroperoxy-3-methyl-6-(prop-1-en-2-yl)cyclohex-1-ene | 21.26 | 16423559 | 0.33 |
|  | 1,2-Cyclohexanediol, 1-methyl-4-(1-methylethenyl)- | 22.49 | 515904037 | 10.35 |
|  | 2-Bornanol, 2-methyl- | 22.96 | 16693762 | 0.33 |
|  | 2,6-Octadien-1-ol, 3,7-dimethyl-, acetate, (Z)- | 23.06 | 55747572 | 1.12 |
|  | 7-Oxabicyclo[4.1.0]heptan-2-one, 3-methyl-6-(1-methylethyl)- | 23.23 | 18030117 | 0.36 |
|  | 2,6-Octadien-1-ol, 3,7-dimethyl-, acetate, (Z)- | 23.88 | 43668528 | 0.88 |
|  | 7-Isopropyl-7-methyl-nona-3,5-diene-2,8-dione | 24.57 | 14623501 | 0.29 |
|  | (1S-(1Alpha,2alpha,4beta))-1-isopropenyl-4-methyl-1,2-cyclohexanediol | 25 | 118060509 | 2.37 |
|  | 6-(3-Hydroxy-but-1-enyl)-1,5,5-trimethyl-7-oxabicyclo[4.1.0]heptan-2-ol | 25.85 | 17683764 | 0.35 |
|  | trans-p-Mentha-2,8-dienol | 26.26 | 19714148 | 0.40 |
|  | (1S-(1Alpha,2alpha,4beta))-1-isopropenyl-4-methyl-1,2-cyclohexanediol | 26.47 | 68384288 | 1.37 |
|  | Bicyclo[3.1.0]hexane-6-methanol, 2-hydroxy-1,4,4-trimethyl- | 26.6 | 4107560 | 0.08 |
|  | 2-Octen-1-ol, 3,7-dimethyl-, isobutyrate, (Z)- | 27.19 | 23628801 | 0.47 |
|  | Sobrerol 8-acetate | 27.64 | 10758856 | 0.22 |
|  | 4,6,6-Trimethyl-bicyclo[3.1.1]heptan-2-ol | 28.36 | 17700223 | 0.36 |
|  | 4-Hexenoic acid, 6-(acetyloxy)-4-methyl- | 29.52 | 14248970 | 0.29 |
|  | 5-(1-Bromo-1-methylethyl)-2-methylcyclohex-2-enone | 34.07 | 15349830 | 0.31 |
|  | (1S-(1Alpha,2alpha,4beta))-1-isopropenyl-4-methyl-1,2-cyclohexanediol | 46.42 | 19815299 | 0.40 |
|  | Sobrerol 8-acetate | 47.4 | 12880541 | 0.26 |
|  | Limonen-6-ol, pivalate | 49.31 | 38164400 | 0.77 |
|  | 4-(2,2,6-Trimethyl-bicyclo[4.1.0]hept-1-yl)-butan-2-one | 51.16 | 33051872 | 0.66 |
|  | Fumagillol | 51.28 | 11959880 | 0.24 |
|  | Limonen-6-ol, pivalate | 51.37 | 10191715 | 0.20 |
|  | Cyclooctatin | 52.14 | 17265831 | 0.35 |
|  | Camphorsulfonic acid | 52.33 | 54524142 | 1.09 |
|  | Propanoic acid, 3-(3-hydroxybicyclo[2.2.1]hept-2-yliden)-2-methyl | 52.38 | 166306195 | 3.34 |
|  | Cyclopropanecarboxylic acid, 2,2-dimethyl-3-(2-methyl-1-propenyl)-, cis- | 52.92 | 77418000 | 1.55 |
|  | Spiro[4.5]decan-7-one, 1,8-dimethyl-8,9-epoxy-4-isopropyl- | 54.15 | 127214725 | 2.55 |
|  | Incensole oxide, methyl ether | 54.69 | 9368956 | 0.19 |
|  | Fumagillol | 55.08 | 53027342 | 1.06 |
|  | Spiro[4.5]decan-7-one, 1,8-dimethyl-8,9-epoxy-4-isopropyl- | 55.53 | 30677742 | 0.62 |
|  | 1H-Benzocyclohepten-7-ol, 2,3,4,4a,5,6,7,8-octahydro-1,1,4a,7-tetramethyl-, cis- | 56.21 | 57927393 | 1.16 |
|  |  |  | 4984641589 | 100.00 |

| black pepper | compound | RT | Area (counts*min) | Relative Height |
| --- | --- | --- | --- | --- |
|  | Bicyclo[3.1.0]hex-2-ene, 4-methyl-1-(1-methylethyl)- | 5.63 | 80905594 | 1.17 |
|  | (1S)-2,6,6-Trimethylbicyclo[3.1.1]hept-2-ene | 5.81 | 40048550 | 0.58 |
|  | 3-Carene | 5.87 | 387701927 | 5.63 |
|  | Ethanone, 1-(3-ethyloxiranyl)- | 6.17 | 27753403 | 0.40 |
|  | Camphene | 6.31 | 16868053 | 0.24 |
|  | Sulfurous acid, butyl isohexyl ester | 6.47 | 21385887 | 0.31 |
|  | Cyclopropane, 1,1,2,2-tetramethyl- | 6.8 | 16196221 | 0.23 |
|  | 2-Pentene, 2,4-dimethyl- | 6.9 | 88463608 | 1.28 |
|  | Bicyclo[3.1.0]hexane, 4-methylene-1-(1-methylethyl)- | 7.21 | 901934462 | 13.09 |
|  | ß-Pinene | 7.64 | 41396126 | 0.60 |
|  | a-Phellandrene | 8.19 | 8680471 | 0.13 |
|  | 3-Carene | 8.29 | 384067076 | 5.57 |
|  | Benzene, 1-methyl-3-(1-methylethyl)- | 8.85 | 81255237 | 1.18 |
|  | Cyclohexene, 1-methyl-5-(1-methylethenyl)-, (R)- | 9.01 | 29166053 | 0.42 |
|  | Cyclohexene, 1-methyl-5-(1-methylethenyl)-, (R)- | 9.05 | 132531788 | 1.92 |
|  | D-Limonene | 9.1 | 334584358 | 4.85 |
|  | 5-Isopropyl-2-methylbicyclo[3.1.0]hexan-2-ol # | 10.58 | 13896339 | 0.20 |
|  | Cyclohexene, 1-methyl-4-(1-methylethylidene)- | 11.23 | 12644009 | 0.18 |
|  | Linalool | 11.87 | 33575363 | 0.49 |
|  | 3-Cyclohexen-1-ol, 4-methyl-1-(1-methylethyl)-, (R)- | 15.18 | 32905143 | 0.48 |
|  | 3-Cyclohexene-1-methanol, a,a,4-trimethyl-, (R)- | 15.82 | 15698649 | 0.23 |
|  | Cyclohexene, 4-ethenyl-4-methyl-3-(1-methylethenyl)-1-(1-methylethyl)-, (3R-trans)- | 21.89 | 246397928 | 3.58 |
|  | a-Cubebene | 22.4 | 50726685 | 0.74 |
|  | 1,2,4-Metheno-1H-indene, octahydro-1,7a-dimethyl-5-(1-methylethyl)-, [1S-(1a,2a,3aß,4a,5a,7aß,8S*)]- | 23.16 | 18330731 | 0.27 |
|  | Copaene | 23.58 | 472092642 | 6.85 |
|  | (1R,2S,6S,7S,8S)-8-Isopropyl-1-methyl-3-methylenetricyclo[4.4.0.02,7]decane-rel- | 24.07 | 13308460 | 0.19 |
|  | Naphthalene, 1,2,3,5,6,7,8,8a-octahydro-1,8a-dimethyl-7-(1-methylethenyl)-, [1R-(1a,7ß,8aa)]- | 24.17 | 157636952 | 2.29 |
|  | Cyclohexane, 1-ethenyl-1-methyl-2,4-bis(1-methylethenyl)-, [1S-(1a,2ß,4ß)]- | 24.22 | 67494337 | 0.98 |
|  | 1H-Cycloprop[e]azulene, 1a,2,3,4,4a,5,6,7b-octahydro-1,1,4,7-tetramethyl-, [1aR-(1aa,4a,4aß,7ba)]- | 24.85 | 30275178 | 0.44 |
|  | Caryophyllene | 25.44 | 1621856680 | 23.53 |
|  | (1R,2S,6S,7S,8S)-8-Isopropyl-1-methyl-3-methylenetricyclo[4.4.0.02,7]decane-rel- | 25.79 | 25477562 | 0.37 |
|  | Di-epi-a-cedrene | 26.03 | 61600732 | 0.89 |
|  | 1,4,7,-Cycloundecatriene, 1,5,9,9-tetramethyl-, Z,Z,Z- | 26.76 | 50488037 | 0.73 |
|  | 1,4,7,-Cycloundecatriene, 1,5,9,9-tetramethyl-, Z,Z,Z- | 26.79 | 96673262 | 1.40 |
|  | cis-ß-Farnesene | 26.92 | 29513377 | 0.43 |
|  | Naphthalene, 1,2,4a,5,8,8a-hexahydro-4,7-dimethyl-1-(1-methylethyl)-, (1a,4aß,8aa)-(±)- | 27.49 | 10850316 | 0.16 |
|  | ?-Muurolene | 27.64 | 17551219 | 0.25 |
|  | (1R,2S,6S,7S,8S)-8-Isopropyl-1-methyl-3-methylenetricyclo[4.4.0.02,7]decane-rel- | 27.8 | 46609487 | 0.68 |
|  | Benzene, 1-(1,5-dimethyl-4-hexenyl)-4-methyl- | 27.95 | 14088748 | 0.20 |
|  | Naphthalene, decahydro-4a-methyl-1-methylene-7-(1-methylethenyl)-, [4aR-(4aa,7a,8aß)]- | 28.13 | 173927034 | 2.52 |
|  | 1H-Cyclopropa[a]naphthalene, 1a,2,3,5,6,7,7a,7b-octahydro-1,1,7,7a-tetramethyl-, [1aR-(1aa,7a,7aa,7ba)]- | 28.22 | 10917398 | 0.16 |
|  | Naphthalene, 1,2,3,4,4a,5,6,8a-octahydro-4a,8-dimethyl-2-(1-methylethenyl)-, [2R-(2a,4aa,8aß)]- | 28.43 | 123172501 | 1.79 |
|  | 1,3-Cyclohexadiene, 5-(1,5-dimethyl-4-hexenyl)-2-methyl-, [S-(R*,S*)]- | 28.53 | 24673068 | 0.36 |
|  | Naphthalene, 1,2,4a,5,6,8a-hexahydro-4,7-dimethyl-1-(1-methylethyl)-, (1a,4aa,8aa)- | 28.6 | 55160404 | 0.80 |
|  | 1H-Benzocycloheptene, 2,4a,5,6,7,8,9,9a-octahydro-3,5,5-trimethyl-9-methylene- | 29.05 | 193346597 | 2.81 |
|  | (3S,3aR,3bR,4S,7R,7aR)-4-Isopropyl-3,7-dimethyloctahydro-1H-cyclopenta[1,3]cyclopropa[1,2]benzen-3-ol | 29.21 | 26240868 | 0.38 |
|  | 1-Isopropyl-4,7-dimethyl-1,2,3,5,6,8a-hexahydronaphthalene | 29.44 | 142240568 | 2.06 |
|  | (E)-1-Methyl-4-(6-methylhept-5-en-2-ylidene)cyclohex-1-ene | 29.76 | 24371805 | 0.35 |
|  | Cyclohexene, 4-[(1E)-1,5-dimethyl-1,4-hexadien-1-yl]-1-methyl- | 30.32 | 10030672 | 0.15 |
|  | Cyclohexanemethanol, 4-ethenyl-a,a,4-trimethyl-3-(1-methylethenyl)-, [1R-(1a,3a,4ß)]- | 30.51 | 41738680 | 0.61 |
|  | 1,6,10-Dodecatrien-3-ol, 3,7,11-trimethyl-, (E)- | 31.15 | 10134137 | 0.15 |
|  | Caryophyllene oxide | 31.68 | 234179150 | 3.40 |
|  | (1R,3E,7E,11R)-1,5,5,8-Tetramethyl-12-oxabicyclo[9.1.0]dodeca-3,7-diene | 32.69 | 13504276 | 0.20 |
|  | Isospathulenol | 33.49 | 29735922 | 0.43 |
|  | 10,10-Dimethyl-2,6-dimethylenebicyclo[7.2.0]undecan-5ß-ol | 33.74 | 12322158 | 0.18 |
|  | 1-Naphthalenol, 1,2,3,4,4a,7,8,8a-octahydro-1,6-dimethyl-4-(1-methylethyl)-, [1R-(1a,4ß,4aß,8aß)]- | 34.17 | 22482323 | 0.33 |
|  | Bicyclo[4.4.0]dec-2-ene-4-ol, 2-methyl-9-(prop-1-en-3-ol-2-yl)- | 34.5 | 11269521 | 0.16 |
|  |  |  | 6892077732 | 100.00 |

| thyme | compound | RT | Area (counts*min) | Relative Height |
| --- | --- | --- | --- | --- |
|  | (1R)-2,6,6-Trimethylbicyclo[3.1.1]hept-2-ene | 5.83 | 85105195 | 1.87 |
|  | Ethanone, 1-(3-ethyloxiranyl)- | 6.16 | 15686409 | 0.35 |
|  | Sulfurous acid, isohexyl 2-propyl ester | 6.47 | 19234640 | 0.42 |
|  | Cyclopropane, 2-bromo-1,1,3-trimethyl- | 6.8 | 18932144 | 0.42 |
|  | Oxalic acid, cyclohexyl pentyl ester | 6.89 | 90067083 | 1.98 |
|  | Bicyclo[3.1.1]heptane, 6,6-dimethyl-2-methylene-, (1S)- | 7.17 | 15745676 | 0.35 |
|  | o-Cymene | 8.88 | 532824700 | 11.73 |
|  | Benzene, 1-methyl-3-(1-methylethyl)- | 8.96 | 184076846 | 4.05 |
|  | Cyclohexene, 1-methyl-5-(1-methylethenyl)-, (R)- | 9.06 | 38204544 | 0.84 |
|  | Eucalyptol | 9.13 | 32702276 | 0.72 |
|  | ?-Terpinene | 10.16 | 86494653 | 1.90 |
|  | 2-Furanmethanol, 5-ethenyltetrahydro-a,a,5-trimethyl-, cis- | 10.63 | 20537475 | 0.45 |
|  | trans-Linalool oxide (furanoid) | 11.28 | 18159691 | 0.40 |
|  | Linalool | 11.98 | 385025316 | 8.48 |
|  | (+)-2-Bornanone | 13.99 | 44102095 | 0.97 |
|  | endo-Borneol | 14.91 | 138385369 | 3.05 |
|  | 3-Cyclohexen-1-ol, 4-methyl-1-(1-methylethyl)-, (R)- | 15.2 | 58833515 | 1.30 |
|  | Terpinen-4-ol | 15.31 | 32474453 | 0.71 |
|  | 3-Cyclohexene-1-methanol, a,a,4-trimethyl-, (R)- | 16.15 | 20910036 | 0.46 |
|  | trans-Ascaridol glycol | 19.14 | 19458332 | 0.43 |
|  | Phenol, 2-methyl-5-(1-methylethyl)- | 20.31 | 2379443171 | 52.39 |
|  | 3-Methyl-4-isopropylphenol | 20.72 | 109872548 | 2.42 |
|  | Caryophyllene | 25.28 | 12117305 | 0.27 |
|  | Caryophyllene oxide | 31.68 | 133858143 | 2.95 |
|  | (1R,3E,7E,11R)-1,5,5,8-Tetramethyl-12-oxabicyclo[9.1.0]dodeca-3,7-diene | 32.69 | 15056634 | 0.33 |
|  | 14-Hydroxycaryophyllene | 35.02 | 23768233 | 0.52 |
|  | 7-[(E)-5-(3-Hydroxy-2,2-dimethyl-6-methylidenecyclohexyl)-3-methylpent-2-enoxy]chromen-2-one acetate | 60.71 | 11110727 | 0.24 |
|  |  |  | 4542187209 | 100.00 |

| geranium | compound | RT | Area (counts*min) | Relative Height |
| --- | --- | --- | --- | --- |
|  | 3-Carene | 4.89 | 4968369 | 0.40 |
|  | Linalool | 9.73 | 33173491 | 2.66 |
|  | (2S,4R)-4-Methyl-2-(2-methylprop-1-en-1-yl)tetrahydro-2H-pyran | 10.55 | 43424438 | 3.48 |
|  | Cyclohexanone, 5-methyl-2-(1-methylethyl)-, (2R-cis)- | 11.78 | 104119633 | 8.34 |
|  | 3-Cyclohexene-1-methanol, a,a,4-trimethyl-, (R)- | 12.81 | 4324353 | 0.35 |
|  | Citronellol | 14.03 | 414003687 | 33.16 |
|  | Geraniol | 14.74 | 62057573 | 4.97 |
|  | (-)-cis-Myrtanol | 15.04 | 4382531 | 0.35 |
|  | 2,6-Octadienal, 3,7-dimethyl-, (E)- | 15.25 | 2982349 | 0.24 |
|  | 6-Octen-1-ol, 3,7-dimethyl-, formate | 15.5 | 153161590 | 12.27 |
|  | 2,6-Octadien-1-ol, 3,7-dimethyl-, formate, (Z)- | 16.28 | 19347597 | 1.55 |
|  | 6-Octen-1-ol, 3,7-dimethyl-, acetate | 17.96 | 7478730 | 0.60 |
|  | Copaene | 18.67 | 6129827 | 0.49 |
|  | (-)-ß-Bourbonene | 18.9 | 14956200 | 1.20 |
|  | Caryophyllene | 20 | 21743571 | 1.74 |
|  | a-Guaiene | 20.54 | 5878186 | 0.47 |
|  | (1R,3aS,8aS)-7-Isopropyl-1,4-dimethyl-1,2,3,3a,6,8a-hexahydroazulene | 20.72 | 85760554 | 6.87 |
|  | 6-Octen-1-ol, 3,7-dimethyl-, propanoate | 20.78 | 12195846 | 0.98 |
|  | (1R,3aS,8aS)-7-Isopropyl-1,4-dimethyl-1,2,3,3a,6,8a-hexahydroazulene | 20.92 | 8984960 | 0.72 |
|  | Humulene | 21.09 | 5533729 | 0.44 |
|  | (1R,9R,E)-4,11,11-Trimethyl-8-methylenebicyclo[7.2.0]undec-4-ene | 21.23 | 4464485 | 0.36 |
|  | (2E,6E)-3,7,11-Trimethyldodeca-2,6,10-trienyl propionate | 21.67 | 14406768 | 1.15 |
|  | (1R,2S,6S,7S,8S)-8-Isopropyl-1-methyl-3-methylenetricyclo[4.4.0.02,7]decane-rel- | 21.88 | 14118057 | 1.13 |
|  | Naphthalene, 1,2,3,5,6,7,8,8a-octahydro-1,8a-dimethyl-7-(1-methylethenyl)-, [1S-(1a,7a,8aa)]- | 22.1 | 5343629 | 0.43 |
|  | 1H-Cycloprop[e]azulene, 1a,2,3,5,6,7,7a,7b-octahydro-1,1,4,7-tetramethyl-, [1aR-(1aa,7a,7aß,7ba)]- | 22.2 | 12383319 | 0.99 |
|  | Naphthalene, 1,2,4a,5,6,8a-hexahydro-4,7-dimethyl-1-(1-methylethyl)-, (1a,4aa,8aa)- | 22.46 | 2932306 | 0.23 |
|  | (S,1Z,6Z)-8-Isopropyl-1-methyl-5-methylenecyclodeca-1,6-diene | 22.86 | 4506653 | 0.36 |
|  | 1-Isopropyl-4,7-dimethyl-1,2,3,5,6,8a-hexahydronaphthalene | 23.05 | 15848743 | 1.27 |
|  | Cyclohexene, 6-ethenyl-6-methyl-1-(1-methylethyl)-3-(1-methylethylidene)-, (S)- | 23.17 | 3573381 | 0.29 |
|  | Citronellyl isobutyrate | 23.36 | 28499447 | 2.28 |
|  | 10,13-Octadecadiynoic acid, methyl ester | 24.09 | 2395836 | 0.19 |
|  | Butanoic acid, 3,7-dimethyl-2,6-octadienyl ester, (E)- | 24.24 | 14096863 | 1.13 |
|  | 1-((1R,2R,3R)-2-(3-Isopropylfuran-2-yl)-3-methylcyclopentyl)ethanone | 24.78 | 11497305 | 0.92 |
|  | 2-Phenylethyl tiglate | 24.91 | 10734881 | 0.86 |
|  | ß-D-Mannofuranoside, farnesyl- | 25.43 | 3359703 | 0.27 |
|  | Cedrol | 25.51 | 4818010 | 0.39 |
|  | 4a(2H)-Naphthalenol, 1,3,4,5,6,8a-hexahydro-4,7-dimethyl-1-(1-methylethyl)-, (1S,4R,4aS,8aR)- | 26.12 | 7569737 | 0.61 |
|  | Citronellyl tiglate | 27.22 | 15961044 | 1.28 |
|  | Pentanoic acid, 4-methyl-, 3,7-dimethyl-6-octenyl ester | 27.78 | 4199689 | 0.34 |
|  | Geranyl tiglate | 28.14 | 18038123 | 1.44 |
|  | Ethanol, 2-(9,12-octadecadienyloxy)-, (Z,Z)- | 28.68 | 6544348 | 0.52 |
|  | Dibutyl phthalate | 34.58 | 3047159 | 0.24 |
|  | Padimate O | 41.48 | 3165180 | 0.25 |
|  | Benzyl butyl phthalate | 42.97 | 16287403 | 1.30 |
|  | Bis(2-ethylhexyl) phthalate | 46.83 | 6158541 | 0.49 |
|  |  |  | 1248557824 | 100.00 |

| Amyris | Library Compound | RT | Area (counts*min) | Relative Height |
| --- | --- | --- | --- | --- |
|  | Damascone, ß- | 24.11 | 31445942 | 0.22 |
|  | 4a,8-Dimethyl-2-(prop-1-en-2-yl)-1,2,3,4,4a,5,6,7-octahydronaphthalene | 24.57 | 16609995 | 0.12 |
|  | Cyclohexane, 1-ethenyl-1-methyl-2,4-bis(1-methylethenyl)-, [1S-(1a,2ß,4ß)]- | 24.74 | 16972254 | 0.12 |
|  | ß-Humulene | 25.24 | 17137261 | 0.12 |
|  | Longifolene-(V4) | 25.45 | 19396547 | 0.14 |
|  | (3R,3aS,8aS)-3,6,8,8-Tetramethyl-2,3,4,7,8,8a-hexahydro-1H-3a,7-methanoazulene | 25.82 | 17641678 | 0.12 |
|  | (4aS,9aR)-3,5,5,9-Tetramethyl-2,4a,5,6,7,9a-hexahydro-1H-benzo[7]annulene | 26.57 | 186618310 | 1.30 |
|  | Spiro[5.5]undec-2-ene, 3,7,7-trimethyl-11-methylene-, (-)- | 26.68 | 39526709 | 0.28 |
|  | cis-Thujopsene | 26.87 | 160632790 | 1.12 |
|  | cis-a-Bergamotene | 26.91 | 338610382 | 2.36 |
|  | (1S,1aS,1bR,4S,5S,5aS,6aR)-1a,1b,4,5a-Tetramethyldecahydro-1,5-methanocyclopropa[a]indene | 27.21 | 24189260 | 0.17 |
|  | (1R,4R,4aS,8aR)-4,7-Dimethyl-1-(prop-1-en-2-yl)-1,2,3,4,4a,5,6,8a-octahydronaphthalene | 27.51 | 110965399 | 0.77 |
|  | Humulane-1,6-dien-3-ol | 27.67 | 78717574 | 0.55 |
|  | (1R,4R,4aS,8aR)-4,7-Dimethyl-1-(prop-1-en-2-yl)-1,2,3,4,4a,5,6,8a-octahydronaphthalene | 27.91 | 396257046 | 2.77 |
|  | (2R,3R,4aR,5S,8aS)-2-Hydroxy-4a,5-dimethyl-3-(prop-1-en-2-yl)octahydronaphthalen-1(2H)-one | 27.94 | 187970483 | 1.31 |
|  | 4a,8-Dimethyl-2-(prop-1-en-2-yl)-1,2,3,4,4a,5,6,7-octahydronaphthalene | 27.99 | 385468747 | 2.69 |
|  | isoledene | 28.08 | 36905517 | 0.26 |
|  | 1-Methyl-4-(6-methylhept-5-en-2-yl)cyclohexa-1,3-diene | 28.27 | 44247654 | 0.31 |
|  | Benzene, 1-(1,5-dimethyl-4-hexenyl)-4-methyl- | 28.5 | 650902980 | 4.55 |
|  | Naphthalene, decahydro-4a-methyl-1-methylene-7-(1-methylethenyl)-, [4aR-(4aa,7a,8aß)]- | 28.67 | 139557511 | 0.97 |
|  | Naphthalene, 1,2,3,5,6,7,8,8a-octahydro-1,8a-dimethyl-7-(1-methylethenyl)-, [1R-(1a,7ß,8aa)]- | 28.83 | 60870061 | 0.43 |
|  | 1,4-Dimethyl-7-(prop-1-en-2-yl)decahydroazulen-4-ol | 28.97 | 92465313 | 0.65 |
|  | 1,3-Cyclohexadiene, 5-(1,5-dimethyl-4-hexenyl)-2-methyl-, [S-(R*,S*)]- | 29.04 | 279158562 | 1.95 |
|  | 2H-3,9a-Methano-1-benzoxepin, octahydro-2,2,5a,9-tetramethyl-, [3R-(3a,5aa,9a,9aa)]- | 29.27 | 136992482 | 0.96 |
|  | Cyclohexane, 1-ethenyl-1-methyl-2-(1-methylethenyl)-4-(1-methylethylidene)- | 29.36 | 67323525 | 0.47 |
|  | ?-Elemene | 29.48 | 16490131 | 0.12 |
|  | ß-Bisabolene | 29.59 | 105999621 | 0.74 |
|  | (3S,3aR,3bR,4S,7R,7aR)-4-Isopropyl-3,7-dimethyloctahydro-1H-cyclopenta[1,3]cyclopropa[1,2]benzen-3-ol | 29.63 | 53815600 | 0.38 |
|  | a-Maaliene | 29.68 | 307478961 | 2.15 |
|  | Cyclohexanemethanol, 4-ethenyl-a,a,4-trimethyl-3-(1-methylethenyl)-, [1R-(1a,3a,4ß)]- | 29.77 | 16885826 | 0.12 |
|  | (1R,3aR,5aR,9aS)-1,4,4,7-Tetramethyl-1,2,3,3a,4,5a,8,9-octahydrocyclopenta[c]benzofuran | 29.89 | 79267257 | 0.55 |
|  | Cyclohexene, 3-(1,5-dimethyl-4-hexenyl)-6-methylene-, [S-(R*,S*)]- | 29.98 | 36794636 | 0.26 |
|  | (1S,5S)-4-Methylene-1-((R)-6-methylhept-5-en-2-yl)bicyclo[3.1.0]hexane | 30.15 | 100674806 | 0.70 |
|  | (1aR,4aS,8aS)-4a,8,8-Trimethyl-1,1a,4,4a,5,6,7,8-octahydrocyclopropa[d]naphthalene-2-carbaldehyde | 30.2 | 198625941 | 1.39 |
|  | 1H-Cyclopropa[a]naphthalene, 1a,2,3,3a,4,5,6,7b-octahydro-1,1,3a,7-tetramethyl-, [1aR-(1aa,3aa,7ba)]- | 30.3 | 37995408 | 0.27 |
|  | (4aR,8aS)-4a-Methyl-1-methylene-7-(propan-2-ylidene)decahydronaphthalene | 30.33 | 81950376 | 0.57 |
|  | Longifolene-(V4) | 30.48 | 117071145 | 0.82 |
|  | Selina-3,7(11)-diene | 30.53 | 191912166 | 1.34 |
|  | (3R,5aS,9aR)-2,2,5a,9-Tetramethyl-3,4,5,5a,6,7-hexahydro-2H-3,9a-methanobenzo[b]oxepine | 30.58 | 234801088 | 1.64 |
|  | 3,7-Cyclodecadiene-1-methanol, a,a,4,8-tetramethyl-, [s-(Z,Z)] | 31.14 | 901151313 | 6.29 |
|  | Cyclohexanemethanol, 4-ethenyl-a,a,4-trimethyl-3-(1-methylethenyl)-, [1R-(1a,3a,4ß)]- | 31.52 | 35232538 | 0.25 |
|  | 3,7-Cyclodecadiene-1-methanol, a,a,4,8-tetramethyl-, [s-(Z,Z)] | 31.99 | 18528585 | 0.13 |
|  | 1-(1-Ethyl-2,3-dimethyl-cyclopent-2-enyl)-ethanone | 32.4 | 28625806 | 0.20 |
|  | 1,6,10-Dodecatrien-3-ol, 3,7,11-trimethyl-, (E)- | 32.54 | 36970270 | 0.26 |
|  | 2-(4a,8-Dimethyl-2,3,4,5,6,8a-hexahydro-1H-naphthalen-2-yl)propan-2-ol | 32.67 | 233374903 | 1.63 |
|  | 2-Naphthalenemethanol, decahydro-a,a,4a-trimethyl-8-methylene-, [2R-(2a,4aa,8aß)]- | 33.06 | 64409563 | 0.45 |
|  | 1H-Indene, 1-ethylideneoctahydro-7a-methyl-, cis- | 33.16 | 133839349 | 0.93 |
|  | Neointermedeol | 33.17 | 42114436 | 0.29 |
|  | 2-((2S,4aR)-4a,8-Dimethyl-1,2,3,4,4a,5,6,7-octahydronaphthalen-2-yl)propan-2-ol | 33.23 | 1063549882 | 7.43 |
|  | 5-Azulenemethanol, 1,2,3,4,5,6,7,8-octahydro-a,a,3,8-tetramethyl-, acetate, [3S-(3a,5a,8a)]- | 33.61 | 125764852 | 0.88 |
|  | 2-((2R,8R,8aS)-8,8a-Dimethyl-1,2,3,4,6,7,8,8a-octahydronaphthalen-2-yl)propan-2-ol | 33.62 | 50252591 | 0.35 |
|  | 2-((2S,4aR)-4a,8-Dimethyl-1,2,3,4,4a,5,6,7-octahydronaphthalen-2-yl)propan-2-ol | 33.74 | 1239743348 | 8.66 |
|  | Hinesol | 34.13 | 75683840 | 0.53 |
|  | Agarospirol | 34.16 | 18917983 | 0.13 |
|  | 5-Azulenemethanol, 1,2,3,4,5,6,7,8-octahydro-a,a,3,8-tetramethyl-, acetate, [3S-(3a,5a,8a)]- | 34.2 | 64643624 | 0.45 |
|  | (-)-Aristolene | 34.41 | 329817123 | 2.30 |
|  | Guaiol | 34.47 | 127498757 | 0.89 |
|  | 2-((2R,8R,8aS)-8,8a-Dimethyl-1,2,3,4,6,7,8,8a-octahydronaphthalen-2-yl)propan-2-ol | 34.49 | 131201797 | 0.92 |
|  | Guaiol | 34.57 | 1065328663 | 7.44 |
|  | 2-((2R,8R,8aS)-8,8a-Dimethyl-1,2,3,4,6,7,8,8a-octahydronaphthalen-2-yl)propan-2-ol | 34.88 | 1755709747 | 12.26 |
|  | 2-((2S,4aR)-4a,8-Dimethyl-1,2,3,4,4a,5,6,7-octahydronaphthalen-2-yl)propan-2-ol | 34.9 | 91921364 | 0.64 |
|  | 7-epi-a-Eudesmol | 35.05 | 307306160 | 2.15 |
|  | (E)-2-((8R,8aS)-8,8a-Dimethyl-3,4,6,7,8,8a-hexahydronaphthalen-2(1H)-ylidene)propan-1-ol | 35.17 | 29281379 | 0.20 |
|  | 10s,11s-Himachala-3(12),4-diene | 35.34 | 99698079 | 0.70 |
|  | 1,5-Naphthalenediol, decahydro-2-(1-hydroxy-1-methylethyl)-4a,8-dimethyl-, 2Ac derivative | 35.49 | 86974362 | 0.61 |
|  | 1,1,7,7a-Tetramethyl-1a,2,6,7,7a,7b-hexahydro-1H-cyclopropa[a]naphthalene | 35.71 | 50623163 | 0.35 |
|  | 1-Naphthalenemethanol, 1,4,4a,5,6,7,8,8a-octahydro-2,5,5,8a-tetramethyl- | 35.77 | 25751295 | 0.18 |
|  | 4-(1,5-Dimethylhex-4-enyl)cyclohex-2-enone | 35.98 | 45250392 | 0.32 |
|  | 1-Naphthalenol, decahydro-1,4a-dimethyl-7-(1-methylethylidene)-, [1R-(1a,4aß,8aa)]- | 36.11 | 81679210 | 0.57 |
|  | Caryophylla-4(12),8(13)-dien-5a-ol | 36.58 | 23418331 | 0.16 |
|  | aR-Curcumen-15-al | 36.75 | 20800758 | 0.15 |
|  | Squamulosone | 36.85 | 36631706 | 0.26 |
|  | 2,6,10-Dodecatrien-1-ol, 3,7,11-trimethyl- | 37.09 | 30191642 | 0.21 |
|  | 7-Tetracyclo[6.2.1.0(3.8)0(3.9)]undecanol, 4,4,11,11-tetramethyl- | 37.45 | 80386635 | 0.56 |
|  | (6R,7R)-Bisabolone | 37.91 | 176573079 | 1.23 |
|  | Phenol, 5-(1,5-dimethyl-4-hexenyl)-2-methyl-, (R)- | 38.12 | 18965508 | 0.13 |
|  | Drim-7-en-11-ol | 38.45 | 398998740 | 2.79 |
|  | (1R,4aR,7R,8aR)-7-(2-Hydroxypropan-2-yl)-1,4a-dimethyldecahydronaphthalen-1-ol | 38.71 | 25612736 | 0.18 |
|  | 3-Isopropyl-6,7-dimethyltricyclo[4.4.0.0(2,8)]decane-9,10-diol | 38.99 | 30382883 | 0.21 |
|  |  |  | 14319153336 | 100.00 |

| Cassia | Library Compound | RT | Area (counts*min) | Relative Height |
| --- | --- | --- | --- | --- |
|  | Benzaldehyde | 6.55 | 113550657 | 1.94 |
|  | Phenylethyl Alcohol | 12.2 | 66871207 | 1.14 |
|  | Benzenepropanal | 14.32 | 51439428 | 0.88 |
|  | 3-Phenylpropanol | 17.31 | 5255231 | 0.09 |
|  | Benzaldehyde, 2-methoxy- | 17.61 | 73675609 | 1.26 |
|  | (Z)-3-Phenylacrylaldehyde | 19.24 | 481365378 | 8.21 |
|  | Cinnamaldehyde, (E)- | 19.5 | 3393025608 | 57.86 |
|  | Coumarin | 25.78 | 382719716 | 6.53 |
|  | Acetic acid, cinnamyl ester | 25.87 | 215577610 | 3.68 |
|  | 2-Propenal, 3-(2-methoxyphenyl)- | 26.5 | 137873392 | 2.35 |
|  | (Z)-2-Methoxycinnamaldehyde | 29.7 | 870443698 | 14.84 |
|  | 6,7-Dimethoxy-2-tetralone | 35.07 | 72042676 | 1.23 |
|  |  |  | 5863840210 | 100.00 |

| patchouli | Library Compound | RT | Area (counts*min) | Relative Height |
| --- | --- | --- | --- | --- |
|  | D-Limonene | 8.93 | 11516411 | 0.22 |
|  | 4,7-Methanoazulene, 1,2,3,4,5,6,7,8-octahydro-1,4,9,9-tetramethyl-, [1S-(1a,4a,7a)]- | 23.79 | 152832819 | 2.88 |
|  | Cyclohexane, 1-ethenyl-1-methyl-2,4-bis(1-methylethenyl)-, [1S-(1a,2ß,4ß)]- | 24.12 | 73118179 | 1.38 |
|  | (1S,1aS,1bR,4S,5S,5aS,6aR)-1a,1b,4,5a-Tetramethyldecahydro-1,5-methanocyclopropa[a]indene | 24.97 | 38863277 | 0.73 |
|  | Caryophyllene | 25.26 | 160829466 | 3.03 |
|  | a-Guaiene | 26.07 | 656216657 | 12.35 |
|  | Seychellene | 26.34 | 381447408 | 7.18 |
|  | Humulene | 26.73 | 28804349 | 0.54 |
|  | 1H-3a,7-Methanoazulene, 2,3,6,7,8,8a-hexahydro-1,4,9,9-tetramethyl-, (1a,3aa,7a,8aß)- | 26.9 | 285856926 | 5.38 |
|  | Valerena-4,7(11)-diene | 27 | 102949427 | 1.94 |
|  | 1H-3a,7-Methanoazulene, octahydro-1,9,9-trimethyl-4-methylene-, (1a,3aa,7a,8aß)- | 27.11 | 59609764 | 1.12 |
|  | Caryophyllene | 27.2 | 16878350 | 0.32 |
|  | a-Guaiene | 27.59 | 20285139 | 0.38 |
|  | Aciphyllene | 28.34 | 218844613 | 4.12 |
|  | Guaia-9,11-diene | 28.53 | 21885913 | 0.41 |
|  | Azulene, 1,2,3,5,6,7,8,8a-octahydro-1,4-dimethyl-7-(1-methylethenyl)-, [1S-(1a,7a,8aß)]- | 28.7 | 808765705 | 15.22 |
|  | a-Maaliene | 29.24 | 16032131 | 0.30 |
|  | (2R,8R,8aS)-8,8a-Dimethyl-2-(prop-1-en-2-yl)-1,2,3,7,8,8a-hexahydronaphthalene | 29.73 | 14010309 | 0.26 |
|  | (1R,4aS,6R,8aS)-8a,9,9-Trimethyl-1,2,4a,5,6,7,8,8a-octahydro-1,6-methanonaphthalen-1-ol | 31.01 | 36778443 | 0.69 |
|  | Tricyclo[5.2.2.0(1,6)]undecan-3-ol, 2-methylene-6,8,8-trimethyl- | 31.15 | 10284008 | 0.19 |
|  | Isoaromadendrene epoxide | 31.32 | 35316648 | 0.66 |
|  | Caryophyllene oxide | 31.59 | 39575374 | 0.74 |
|  | 2-(4a,8-Dimethyl-1,2,3,4,4a,5,6,7-octahydro-naphthalen-2-yl)-prop-2-en-1-ol | 32.3 | 63870540 | 1.20 |
|  | Tricyclo[5.2.2.0(1,6)]undecan-3-ol, 2-methylene-6,8,8-trimethyl- | 32.92 | 17969183 | 0.34 |
|  | Longipinocarveol, trans- | 33.03 | 30326435 | 0.57 |
|  | 2-Naphthalenol, 2,3,4,4a,5,6,7-octahydro-1,4a-dimethyl-7-(2-hydroxy-1-methylethyl) | 33.31 | 12713415 | 0.24 |
|  | (3aR,4R,7R,7aS)-1,1,3a,7-tetramethyl-2,3,4,5,6,7,7a,7b-octahydro-1aH-cyclopropa[a]naphthalen-4-ol | 33.42 | 55496325 | 1.04 |
|  | Ledene oxide-(II) | 33.52 | 16295221 | 0.31 |
|  | 1,4-Dimethyl-7-(prop-1-en-2-yl)decahydroazulen-4-ol | 34.62 | 187935562 | 3.54 |
|  | Patchouli alcohol | 34.86 | 1556129358 | 29.28 |
|  | Aristolene epoxide | 35.24 | 16533688 | 0.31 |
|  | (3S,5R,8S)-3,8-Dimethyl-5-(prop-1-en-2-yl)-2,3,5,6,7,8-hexahydroazulen-1(4H)-one | 36.12 | 26554516 | 0.50 |
|  | 4-Hydroxy-6-methyl-3-(4-methylpentanoyl)-2H-pyran-2-one | 36.59 | 84227314 | 1.58 |
|  | Bicyclo[4.4.0]dec-2-ene-4-ol, 2-methyl-9-(prop-1-en-3-ol-2-yl)- | 36.8 | 12567118 | 0.24 |
|  | Longiverbenone | 37.13 | 15597874 | 0.29 |
|  | (3aR,4R,7R,7aS)-1,1,3a,7-tetramethyl-2,3,4,5,6,7,7a,7b-octahydro-1aH-cyclopropa[a]naphthalen-4-ol | 37.24 | 15403498 | 0.29 |
|  | Corymbolone | 42.31 | 12243907 | 0.23 |
|  |  |  | 5314565270 | 100.00 |

| juniper berry | Library Compound | RT | Area (counts*min) | Relative Height |
| --- | --- | --- | --- | --- |
|  | Bicyclo[3.1.0]hex-2-ene, 4-methyl-1-(1-methylethyl)- | 5.52 | 14468471 | 0.65 |
|  | Bicyclo[3.1.1]hept-2-ene, 3,6,6-trimethyl- | 5.65 | 16968424 | 0.77 |
|  | 3-Carene | 5.79 | 802093199 | 36.27 |
|  | (1R)-2,6,6-Trimethylbicyclo[3.1.1]hept-2-ene | 6.02 | 6079728 | 0.27 |
|  | (1S)-2,6,6-Trimethylbicyclo[3.1.1]hept-2-ene | 6.12 | 5586086 | 0.25 |
|  | Bicyclo[3.1.0]hexane, 4-methylene-1-(1-methylethyl)- | 7 | 379040109 | 17.14 |
|  | Bicyclo[3.1.1]heptane, 6,6-dimethyl-2-methylene-, (1S)- | 7.58 | 230004095 | 10.40 |
|  | o-Cymene | 8.75 | 48243272 | 2.18 |
|  | Cyclohexene, 1-methyl-5-(1-methylethenyl)-, (R)- | 8.93 | 118928811 | 5.38 |
|  | ?-Terpinene | 10.06 | 25652300 | 1.16 |
|  | Cyclohexene, 3-methyl-6-(1-methylethylidene)- | 11.15 | 22637379 | 1.02 |
|  | 3-Cyclohexen-1-ol, 4-methyl-1-(1-methylethyl)-, (R)- | 15.13 | 75797650 | 3.43 |
|  | a-Cubebene | 22.34 | 10697842 | 0.48 |
|  | Cyclohexane, 1-ethenyl-1-methyl-2,4-bis(1-methylethenyl)-, [1S-(1a,2ß,4ß)]- | 24.11 | 21902912 | 0.99 |
|  | Caryophyllene | 25.25 | 127763132 | 5.78 |
|  | ?-Elemene | 25.76 | 28353666 | 1.28 |
|  | 1,4,7,-Cycloundecatriene, 1,5,9,9-tetramethyl-, Z,Z,Z- | 26.69 | 98277592 | 4.44 |
|  | ?-Muurolene | 27.56 | 11633369 | 0.53 |
|  | (1R,2S,6S,7S,8S)-8-Isopropyl-1-methyl-3-methylenetricyclo[4.4.0.02,7]decane-rel- | 27.74 | 52720808 | 2.38 |
|  | Naphthalene, 1,2,4a,5,6,8a-hexahydro-4,7-dimethyl-1-(1-methylethyl)-, (1a,4aa,8aa)- | 28.53 | 10554706 | 0.48 |
|  | ?-Muurolene | 29.05 | 14426014 | 0.65 |
|  | 1-Isopropyl-4,7-dimethyl-1,2,3,5,6,8a-hexahydronaphthalene | 29.32 | 45532968 | 2.06 |
|  | ?-Elemene | 30.73 | 12298869 | 0.56 |
|  | Caryophyllene oxide | 31.58 | 15526475 | 0.70 |
|  | m-Camphorene | 44.58 | 16180270 | 0.73 |
|  |  |  | 2211368147 | 100.00 |

| clove | Library Compound | RT | Area (counts*min) | Relative Height |
| --- | --- | --- | --- | --- |
|  | eugenol | 22.97 | 1077752494 | 86.51 |
|  | Caryophyllene | 25.43 | 118964404 | 9.55 |
|  | 1,4,7,-Cycloundecatriene, 1,5,9,9-tetramethyl-, Z,Z,Z- | 26.76 | 16538350 | 1.33 |
|  | 3-Allyl-6-methoxyphenyl acetate | 29.31 | 15792410 | 1.27 |
|  | 14-Hydroxycaryophyllene | 31.65 | 16783243 | 1.35 |
|  |  |  | 1245830901 | 100.00 |

| citronella | Library Compound | RT | Area (counts*min) | Relative Height |
| --- | --- | --- | --- | --- |
|  | D-Limonene | 8.91 | 27905463 | 4.46 |
|  | (1R,2R,5S)-5-Methyl-2-(prop-1-en-2-yl)cyclohexanol | 13.73 | 17220428 | 2.75 |
|  | 6-Octenal, 3,7-dimethyl-, (R)- | 14.02 | 106293355 | 17.00 |
|  | 6-Octenal, 3,7-dimethyl-, (R)- | 14.05 | 158008568 | 25.27 |
|  | (1R,2R,5S)-5-Methyl-2-(prop-1-en-2-yl)cyclohexanol | 14.16 | 6297278 | 1.01 |
|  | Citronellol | 17.31 | 79854305 | 12.77 |
|  | Geraniol | 18.31 | 53701046 | 8.59 |
|  | Geraniol | 18.34 | 68281649 | 10.92 |
|  | Eugenol | 22.5 | 6029750 | 0.96 |
|  | 6-Octen-1-ol, 3,7-dimethyl-, acetate | 22.59 | 17719368 | 2.83 |
|  | Geranyl acetate | 23.79 | 18779823 | 3.00 |
|  | Cyclohexane, 1-ethenyl-1-methyl-2,4-bis(1-methylethenyl)-, [1S-(1a,2ß,4ß)]- | 24.1 | 14889164 | 2.38 |
|  | 1-Isopropyl-4,7-dimethyl-1,2,3,5,6,8a-hexahydronaphthalene | 29.3 | 13418104 | 2.15 |
|  | 3,7-Cyclodecadiene-1-methanol, a,a,4,8-tetramethyl-, [s-(Z,Z)] | 30.44 | 27173541 | 4.35 |
|  | (2E,4S,7E)-4-Isopropyl-1,7-dimethylcyclodeca-2,7-dienol | 31.44 | 9681047 | 1.55 |
|  |  |  | 625252889 | 100.00 |

| peppermint | Library Compound | RT | Area (counts*min) | Relative Height |
| --- | --- | --- | --- | --- |
|  | Bicyclo[3.1.0]hexane, 4-methylene-1-(1-methylethyl)- | 7.05 | 15344711 | 0.55 |
|  | Bicyclo[3.1.1]heptane, 6,6-dimethyl-2-methylene-, (1S)- | 7.18 | 37357388 | 1.35 |
|  | Cyclohexene, 1-methyl-5-(1-methylethenyl)-, (R)- | 8.89 | 1758287 | 0.06 |
|  | D-Limonene | 8.98 | 55475011 | 2.00 |
|  | Eucalyptol | 9.16 | 199342928 | 7.18 |
|  | Cyclohexanone, 5-methyl-2-(1-methylethyl)-, (2R-cis)- | 14.21 | 796549721 | 28.71 |
|  | Benzofuran, 4,5,6,7-tetrahydro-3,6-dimethyl- | 14.47 | 8024668 | 0.29 |
|  | Cyclohexanone, 5-methyl-2-(1-methylethyl)-, (2R-cis)- | 14.55 | 180888856 | 6.52 |
|  | Cyclohexanol, 1-methyl-4-(1-methylethyl)- | 15.15 | 1132995135 | 40.83 |
|  | Cyclohexanone, 5-methyl-2-(1-methylethylidene)- | 17.65 | 28586149 | 1.03 |
|  | 3-Cyclohexen-1-one, 2-isopropyl-5-methyl- | 18.31 | 11638034 | 0.42 |
|  | Cyclohexanol, 5-methyl-2-(1-methylethyl)-, acetate, (1a,2ß,5ß)- | 19.85 | 43738306 | 1.58 |
|  | (1S)-(+)-Neomenthyl acetate | 20.04 | 151607757 | 5.46 |
|  | Caryophyllene | 25.27 | 96791866 | 3.49 |
|  | (1R,2S,6S,7S,8S)-8-Isopropyl-1-methyl-3-methylenetricyclo[4.4.0.02,7]decane-rel- | 27.76 | 14787756 | 0.53 |
|  |  |  | 2774886573 | 100.00 |
